# Amplified chiroptic response in a multi-helical penta-perylene structure

**DOI:** 10.1039/d6sc03791g

**Published:** 2026-06-17

**Authors:** Stephan K. Pedersen, Michael L. Steigerwald, Colin Nuckolls, Michael Pittelkow

**Affiliations:** a Department of Chemistry, Columbia University New York 10027 USA cn37@columbia.edu; b Department of Chemistry, University of Copenhagen Universitetsparken 5 Copenhagen Ø DK-2100 Denmark pittel@chem.ku.dk

## Abstract

We report the synthesis of a 29-ring, multi-helical penta-perylene (diNPDH) that displays ECD across most of the visible spectrum and a *g*-factor ∼4*x* that of its mono-helicene parent. This electron deficient multi-helical penta-perylene exhibits an amplified, broadband chiroptical response across the visible spectrum. This unique structure was prepared by the fusion of perylene and perylenediimide (PDI). This heavily contorted nano-carbon molecule can be viewed as either a penta-perylene, a twice annulated double [6]helicene, a dimer of π-extended [7]helicenoids or as a centrally-annulated, cross-conjugated [7]twistacene. The chirality of the structure is induced by the helical units, and the electrochemistry is dominated by the PDI units. The molecule exists in three distinct, conformationally stable stereoisomers, all of which are accessible. This unique structure is electroactive and readily accepts eight electrons, one per imide.

## Abstract 2

An electron deficient multi-helical penta-perylene with an amplified, broadband chiroptical response was prepared by the fusion of perylene and perylenediimide (PDI). This contorted nano-carbon contains 29 fused benzene rings.

## Graphical abstract

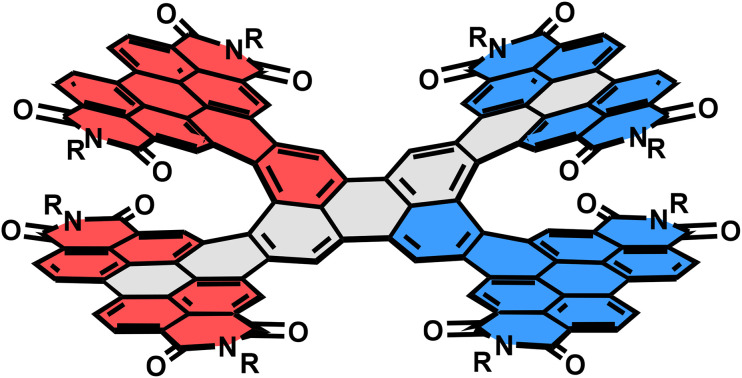

Chiral molecules and materials enable applications ranging from chiroptical switches,^1^ circularly polarized luminescence,^2^ nonlinear optics^3^ to spin filters.^4^ Helicenes are chiral aromatic molecules that interact strongly with circular polarized light, and as a result give intense signals in electronic circular dichroism (ECD) spectroscopy.^5^ While significant attention has been devoted to synthesis of long carbo[*n*]helicenes and hetero[*n*]helicenes, strategies for elongation of these compounds (higher *n*) do little to shift absorption into the visible spectrum or to enhance the affinity towards circularly polarized light beyond *n* = 7.^6^ The last decade has witnessed a renaissance in the studies of helicene structures, and the syntheses of multiple helicenes have expanded the chemical space of helicenes.^7^ Recently, a series of helical structures with multiple helicenes incorporating perylene diimide (PDI) units with large ECD response in the visible part of the optical spectrum was described.^8^ The origin of chiroptical amplification in helical conjugated molecules is poorly understood due to the limited number of small-molecule helicenes that combine strong dissymmetry (|*g*| > ∼10^−3^) with broadband ECD that extends across the visible spectrum—the threshold of “enhanced” chiroptical response targeted herein.^9^

Helicenes are promising chiral materials due to their helical path for π-conjugation.^10,11^ Moreover, the chiroptic properties of helicenes can be enhanced by supramolecular aggregation, photoswitching, reduction/oxidation, and changes in pH.^12^ However, it is a significant challenge to amplify the chiroptic properties of helicenes. Common strategies such as elongating the helical framework, expanding helicenes laterally, fusing multiple helicenes together, or including other heteroatoms in the helical framework typically yield only modest enhancements in chiroptic response, particularly in the visible region of the spectrum.^13–16^ The enhanced absorption of circular polarized light does not appear in other examples of helicenes incorporating PDI- or PDI-like units.^17,18^ In contrast, nano-sized helices display large ECD when they are connected with conjugated linkers.^19^ Here we design a multihelicene based PDI material with enhanced ECD.^20^ We report a simple gram scale preparation of this novel multihelical PDI tetramer. It features intense absorptivity in the visible spectrum with a corresponding high affinity for circularly polarized light.

During studies of PDI oligomers, we have reported on a naphthyl linked PDI dimer helicene (NPDH).^21^ Within the carbon skeleton of NPDH is a non-racemizable carbo[6]helicene motif (outlined in grey, Fig. 1a). NPDH has been used as the synthetic seed and inspiration for the synthesis and characterization of a series of extended or elongated PDI based helicenes, having impressive chiroptical properties.^22^ Systematic variation of the helical framework is needed to identify which design features drive chiroptical amplification. Herein we report on a different strategy to extend the π-system of NPDH (Fig. 1). These new compounds are formally naphthyl fused dimers of NPDH, therefore we denote these novel multihelicenes as diNPDH. This dimerization gives an expanded double carbo[6]helicene motif which is connected by one benzene ring (highlighted in grey, Fig. 1). Given the recently introduced nomenclature this can be defined as an [E_1_]-type double carbo[6]helicene, which notably has never been reported before.^23^

**Fig. 1:**
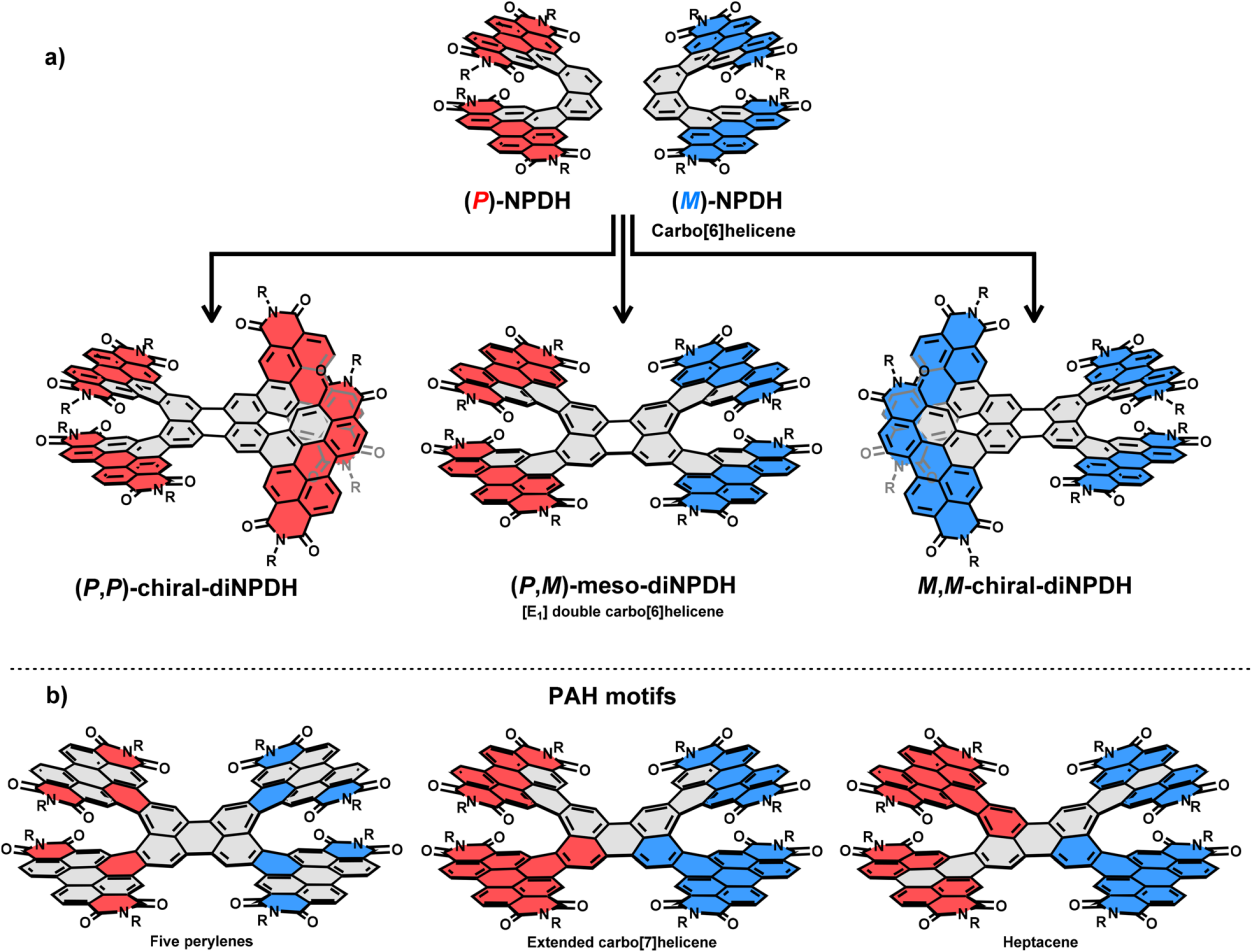
(a) Fusions of the enantiomers of NPDH to give the three isomers of diNPDH. (b) Additional PAH motifs in diNPDH illustrated for *meso*-diNPDH.

The intrinsic chiral nature of NPDH, manifests in the formation of three stereoisomers of diNPDH; a set of enantiomers and a *meso* isomer. Connecting two NPDHs of the same handedness gives either (*M*)- or (*P*)-chiral-diNPDH. Chiral-diNPDH is shaped as a *C*_2_ symmetrical four bladed propeller, with each propeller either tilted left ((*P*)-chiral-diNPDH) or right ((*M*)-chiral-diNPDH). In the scenario where (*M*)-NPDH is connected with (*P*)-NPDH, it will give a butterfly shaped, *C*_4_ symmetric and *meso* isomeric compound denoted as *meso*-diNPDH.

Besides the double carbo[6]helicene motif, a series of other distinct polyaromatic hydrocarbon motifs are present in diNPDH (Fig. 1b). Five fused perylenes are present, forming the overall carbon skeleton, containing a total of 29 annulated benzene rings. Spanning the bay region of the central perylene a π-extended carbo[7]helicene/dibenzo[*a*,*o*]pentaphene is observed, in principle introducing two additional stereogenic axes in diNPDH as a consequence of the helical chirality. Due to the symmetric nature of diNPDH two dibenzo[*a*,*o*]pentaphene fragments are present, and as this motif constitutes half of the kekulene structure, this PAH framework of diNPDH can be viewed as inside-out kekulene.^23^ Looking across the perylene core one of the two heptacene motifs is identified, which due to the chiral nature of diNPDH can be considered a twistacene. This motif is in itself unstable, but when embedded in a PDI framework excellent chemical stability is achieved. Each of these different structural features contributes to the properties of the molecule; the PDI units dominate the electrochemical behavior and the twisted nature contributes to the chiral-optical output.

With the structural rationale in hand, we designed a synthesis that exploits the symmetry of the dimer of NPDH. The latter was prepared in near quantitative yields from 2,7-diBpin-napthalane and 1-bromo-PDI (1), *via* double Suzuki coupling followed by a light mediated regioselective oxidative photocyclization.^24^ Using the appropriate dimer of the naphthalene core, *i.e.* perylene, provides a simple synthetic route to diNPDH (Scheme 1).

**Scheme 1:**
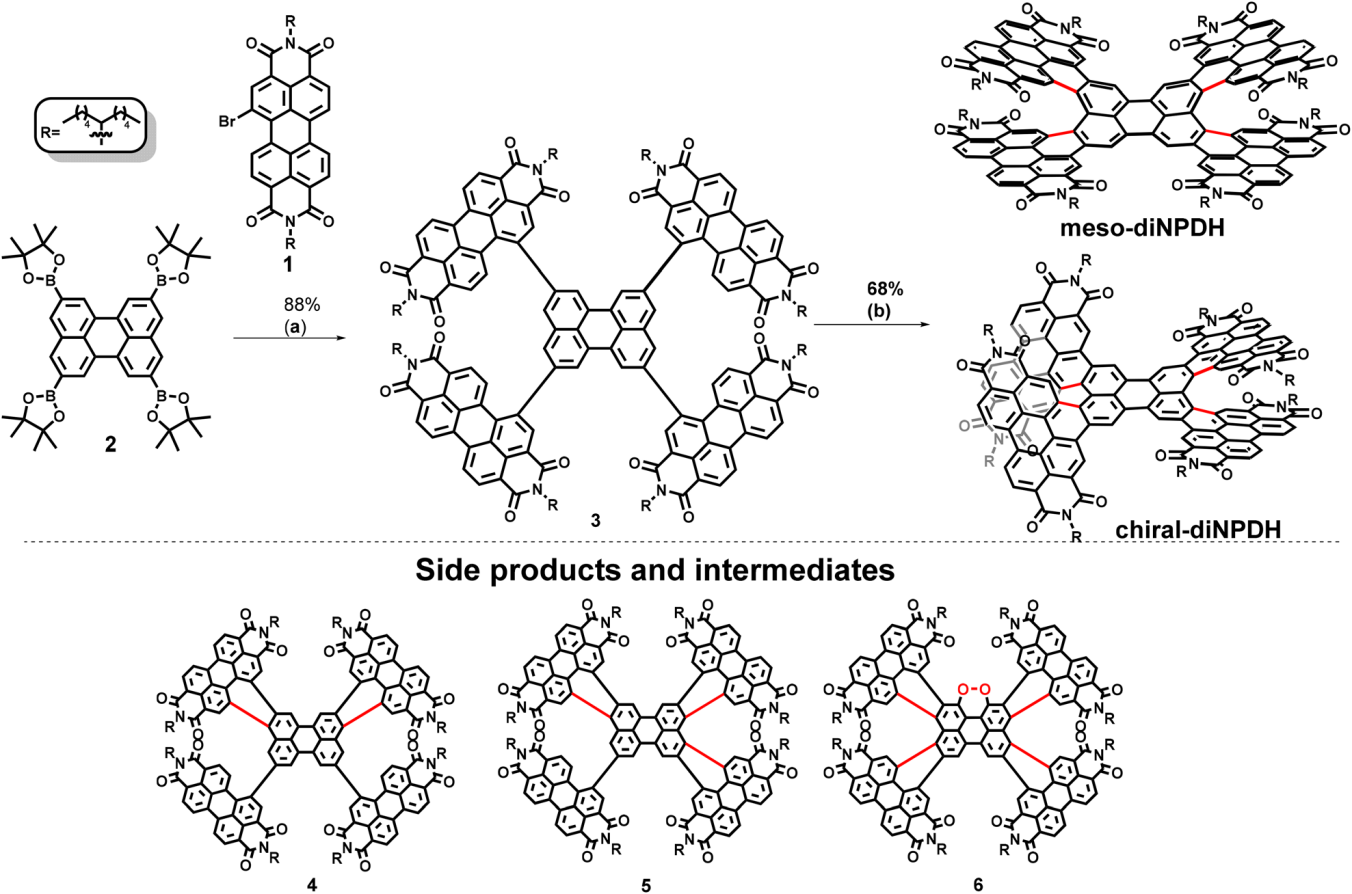
Synthesis of diNPDH. Reagents and conditions: (a) 1-bromo-PDI (4.4 eq.), Pd(dppf)Cl_2_ (0.40 eq.), K_2_CO_3_ (80 eq.), THF : H_2_O (7 : 3), 50 °C, 18 h, 88% (b) FeCl_3_/EtNO_2_, chlorobenzene, 100 °C, 18 h, 68%.

Modification of literature conditions allowed for the chromatography free and gram scale preparation of tetra-borylated perylene 2 (see the SI for details).^25^ Subsequent fourfold Suzuki coupling of 2 with 1-bromo-PDI (1), gave the target tetra-cross coupled product 3 in an excellent yield as a mixture of atropoisomers. With the uncyclized tetramer in hand, attention was turned to exploration of the fourfold cyclodehydrogenation that would furnish the target diNPDH. Initial attempt for the preparation of diNPDH, was the oxidative photocyclization of 3 using 2 × 55 W Compact Fluorescent Light (CFL) bulbs and iodine as the oxidant, a protocol which furnished NPDH in quantitative yield. Unfortunately, even with increased temperature and prolonged reaction times, only double cyclized tetramer 4 was observed as indicated by MALDI-TOF analysis. In order to force the photocyclization to completion, it was investigated by cycling in a visible light flow reactor. The mass of diNPDH was satisfactorily detected by MALDI-TOF analysis, but formation of oxygenated product(s) 6 hindered the isolation of diNPDH. The oxygenated products formed are attributed to leakage of oxygen into the system and generation of reactive oxygen species (ROS), likely singlet oxygen, with diNPDH, or one of the partially cyclized intermediates, acting as the photosensitizer. Thus, the balance between the necessity of oxygen as an oxidant for the C–C forming reaction and the tendency to form over-oxidized products was counter-productive. Accepting that photocyclization would not give the target product, focus was turned to the Scholl reaction for the exhaustive cyclodehydrogenation of 3 to form the target diNPDH. The FeCl_3_/EtNO_2_ system has previously proven successful for the ring fusion of the bay position of PDI with electron-rich aromatic systems.^26^ The mass of the triple cyclized product solely obtained by subjecting the uncyclized tetramer to excess of FeCl_3_ and EtNO_2_ in dichloromethane at room temperature was detected by MALDI-TOF analysis. It should be noted that for entries 1, 2 and 4, analysis of the MALDI-TOF spectra indicates complete selectivity in the number of cyclodehydrogenation*, i.e.* it appears possible to precisely dictate the number of cyclizations by simply varying the reaction conditions. Isolation and analysis of the obtained product(s) nonetheless proved futile, likely due to atropoisomers with different isoenergetic conformations. Increasing the temperature to 100 °C and using chlorobenzene as the solvent, quantitative conversion solely to diNPDH was satisfactorily observed by ^1^H-NMR spectroscopy and MALDI-TOF analysis, with no detectable sign of any side products. The combined isolated yield of the two diastereomers after preparative HPLC purification was 68%, with 44% being the chiral-diNPDH and 24% being the *meso*-diNPDH.

An added advantage of the Scholl reaction, compared to the traditional photocyclization conditions, is ease in scalability. Scaling photocyclizations is hindered by the high dilutions required and/or long reaction times. The diNPDH could readily be prepared on a 1.2 gram scale using only 50 mL of solvent.

Investigation of the ^1^H-NMR spectrum of the unpurified reaction mixture revealed a 2.3 : 1.0 ratio of chiral-diNPDH and *meso*-diNPDH. As the expected statistical distribution is 1 : 1 (*meso*-diNPDH : chiral-diNPDH), the propeller conformation is favored under the present reaction conditions. This is consistent with the general trend observed with multiple helicenes, *i.e.* the propeller conformation is generally the thermodynamically favored product.^25^

The two diastereomers of diNPDH (chiral-diNPDH and *meso*-diNPDH) were isolated in 44% and 24% yield, respectively. The assignment of conformers was based on chiral HPLC analysis, *i.e.* the compound featuring two peaks in the chromatogram is assigned to chiral-diNPDH, and the compound with only one is consistent with *meso*-diNPDH (Fig. 2a). The total yield of diNPDH was only 68%, but investigation of the ^1^H-NMR of the reaction mixture indicates almost quantitative conversion to chiral-diNPDH and *meso*-diNPDH (Fig. 2b), with the lower yield attributed to loss during the preparative HPLC purification protocol.

**Fig. 2:**
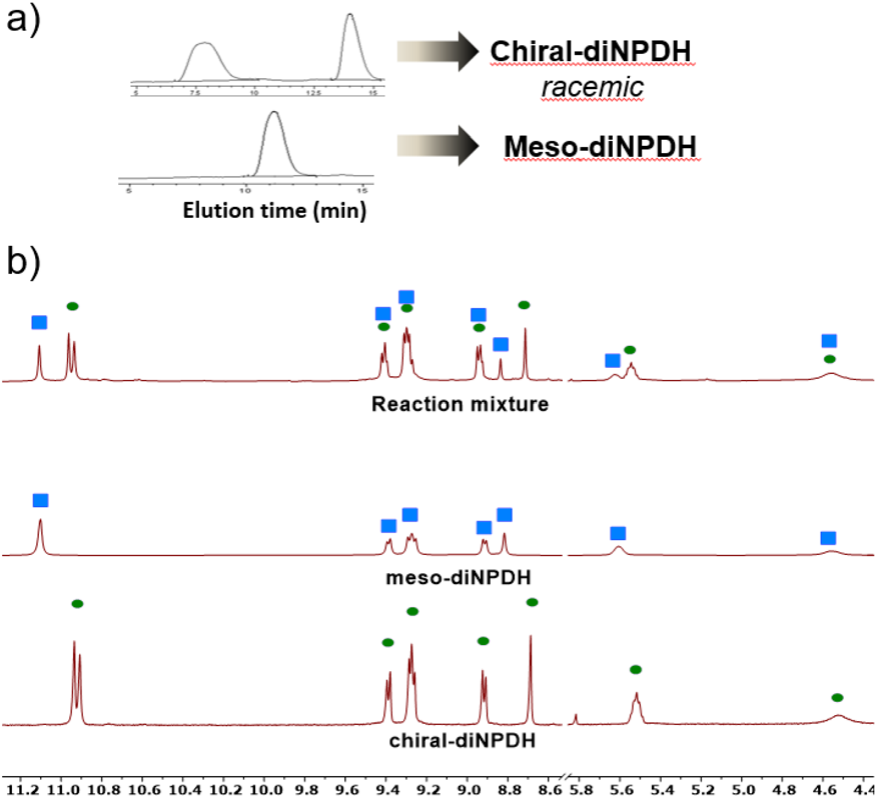
(a) Identification of chiral-diNPDH and *meso*-diNPDH*via* chiral HPLC. (b) ^1^H-NMR (C_2_D_2_Cl_4_ at 370 K) of the reaction mixture, chiral-diNPDH and *meso*-diNPDH recorded in C_2_D_2_Cl_4_ at 370 K.

From the highly symmetrical ^1^H-NMR spectra of *meso*-diNPDH and chiral-diNPDH (see the SI for full spectra), it can be concluded that the oxidative cyclization proceeds with complete regioselectivity. Numerous attempts to grow crystals suitable for SCXRD of either diastereoisomers of diNPDH proved unsuccessful. We have assigned the structure of diNPDH as shown, based on reactivity and NMR considerations. First, the cyclodehydrogenation is done *via* a Scholl reaction, where the selectivity is towards the most electron rich position of the aromatic unit.^27^ It is well-established that electrophilic aromatic substitution, *e.g.* Friedel–Crafts acylation, nitration and bromination, proceeds exclusively at the 3-position of perylene.^27^ Hence the observed reactivity of pristine perylene dictates the selectivity in the cyclodehydrogenation to be exclusively at the *peri*-positions. Secondly, analysis of the ^1^H-NMR spectra of *meso*-diNPDH and chiral-diNPDH bears a striking resemblance to NPDH. Upon heating either of the diastereomers of diNPDH for 2 hours at 250 °C in diphenylether no interconversion between the conformations was observed by HPLC analysis.^28^

Insights into the electronic structure of chiral-diNPDH and *meso*-diNPDH came from their UV/Vis absorption spectra and TD-DFT calculations. For reference the obtained data are compared to those of NPDH. The diastereomers of diNPDH have practically identical absorption spectra in THF (Fig. 3). Furthermore, the experimentally observed absorption spectrum is comparable to what was predicted by TD-DFT calculations (Fig. 4). The main spectral features are intense absorption between 400 and 550 nm, with *λ* at 475 nm (*ε* = 1.5 × 10^5^ M^−1^ cm^−1^) and a shoulder peak of similar absorptivity at 510 nm (*ε* = 1.3 × 10^5^ M^−1^ cm^−1^). These peak maxima occur at similar wavelengths to those observed for NPDH, but with a shift in relative intensities (*ε*_475 nm_ = 0.57 × 10^5^ M^−1^ cm^−1^, *ε*_510 nm_ = 0.73 × 10^5^ M^−1^ cm^−1^). An approximate three-fold increase in absorptivity is noted for the transition at 475 nm. This multifold increase in molar absorptivity at peak maxima is consistent with what has previously been observed for symmetrical double helicenes, relative to their monohelical moiety.^8,9^ Comparing the molar absorptivity of diNPDH to that of the linearly annulated PDIs it is noted that the absorptivities of the two complement each other perfectly. The latter are characterized by low molar absorptivity in the 400–550 nm region and high molar absorptivity in the flanking regions, the opposite as observed for the diNPDHs.

**Fig. 3:**
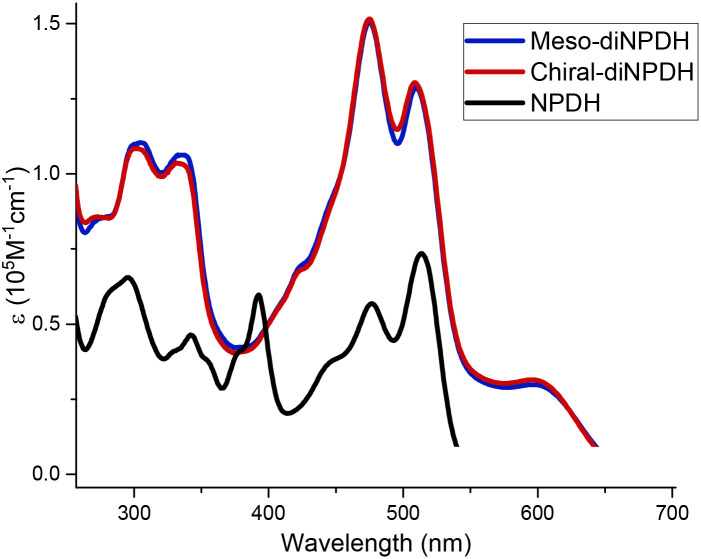
Molar absorbance spectra of *meso*-diNPDH, chiral-diNPDH and diNPDH recorded in THF.

**Fig. 4:**
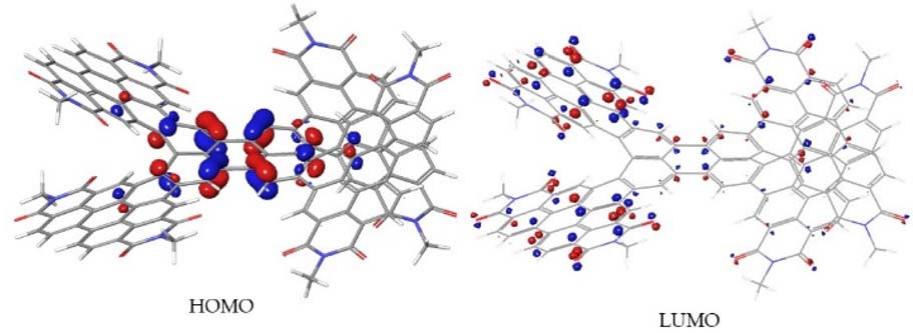
Calculated HOMO and LUMO for chiral-diNPDH.

A major qualitative difference between the absorption spectra of NPDH and diNPDH is the broad lowest energy absorption band (*λ* = ∼600 nm) of diNPDH. TD-DFT calculations were used to identify the origins of this HOMO–LUMO transition. The HOMO was found to be localized across the electron-rich perylene core, and the LUMO to be centered primarily across the electron deficient PDI units (Fig. 4). The feature at ∼600 nm is due to the HOMO–LUMO transition, where the HOMO is localized on the internal perylene hub and the LUMO is distributed among the four peripheral PDI blades. Although the diNPDH framework is pseudo-centrosymmetric, the helical distortion at the bay regions breaks the formal inversion symmetry and renders the HOMO → LUMO transition allowed; TD-DFT predicts a finite oscillator strength for this transition (see SI Fig. S5), consistent with the moderately intense ICT band observed experimentally at ∼600 nm.

Hence, the broad band at 604 nm can be assigned to an intramolecular charge transfer (ICT) band between the electron rich perylene core and the electron poor PDI subunits. Similar observation has previously been reported for anthracenyl linked PDIs.^8,9^

The enantiomers of chiral-diNPDH were isolated using preparative chiral HPLC (see the SI for details). The first eluting peak is denoted as chiral-diNPDH-1 and the second eluting peak is denoted as chiral-diNPDH-2. Their ECD spectra were recorded in THF. The wealth of electronic transition present in chiral-diNPDH is manifested in the CD-spectrum with a series of distinct Cotton effects. Between 260–313 nm and 460–512 nm, preferential absorption of light of one handedness is observed, and in the regions 313–460 nm and 512–640 nm, absorption of light of the opposite handedness is observed. This means that chiral-diNPDH has ECD response across the majority of the visible spectrum. The highest ECD is observed at 525 nm (Δ*ε* = 400 M^−1^ cm^−1^), with a related pronounced Cotton effect, surpassing previous PDI-carbo[6]helicene compounds.^22^ Notably this high ECD response in the 500–550 nm region complements other PDI helicenes, where the highest ECD response is generally in the 400–450 nm region, underscoring the welcome addition of chiral-diNPDH to the PDI helicene family (see Fig. S6 in the SI for structural comparison).^9^ Relative to NPDH, an increase in ECD response is observed across the entire spectrum. To decouple if this increase in ECD response (Δ*ε*) is simply a consequence of chiral-diNPDH having a higher *ε* we plot the *g*-factor (|Δ*ε*|/*ε*, Fig. 5). The notable difference between the *g*-factor of the two substrates is in the 325–400 nm region, where chiral-diNPDH has an approximately fourfold increase in the *g*-factor, compared to NPDH at the local maxima (0.001 *vs.* 0.004, respectively).

**Fig. 5:**
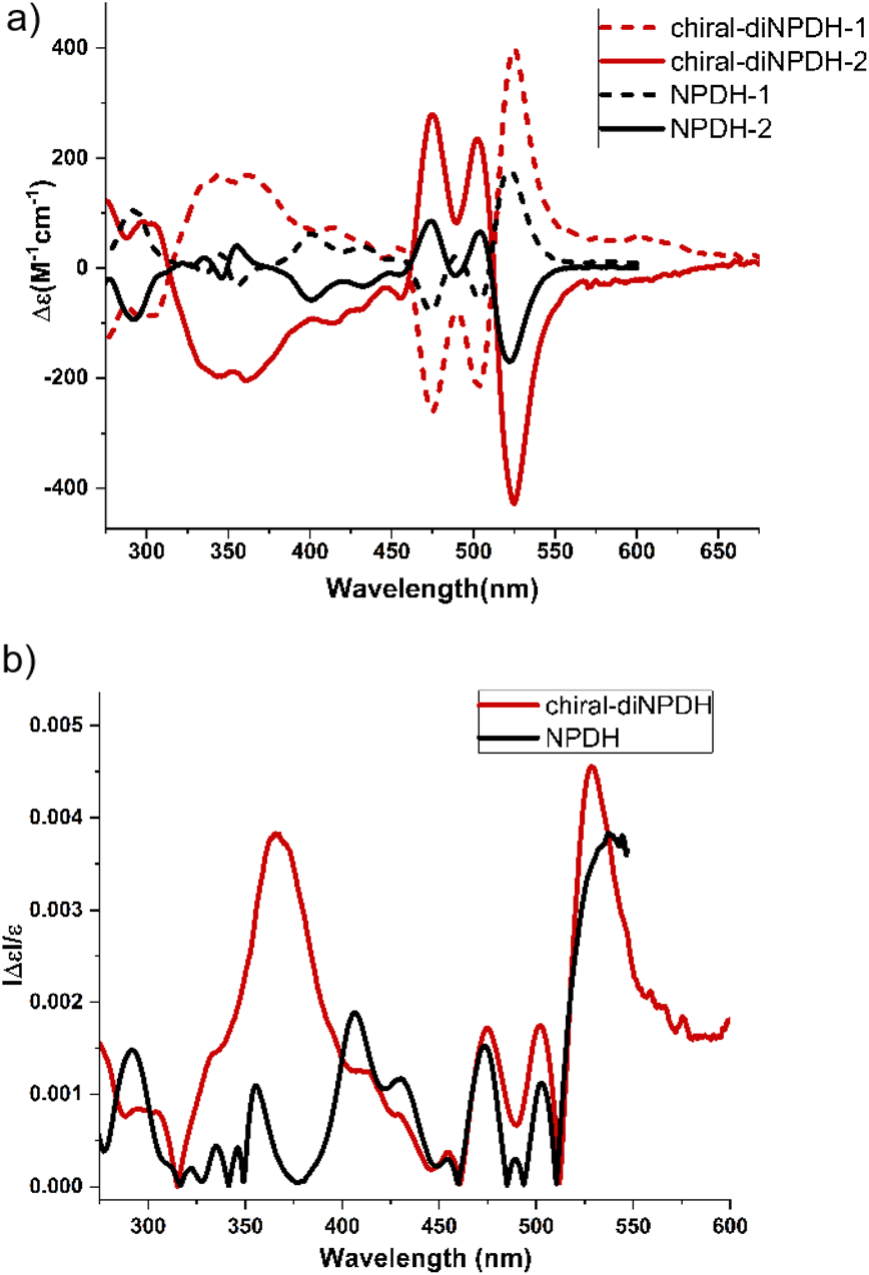
(A) Molar CD-spectrum of the isolated enantiomers of diNPDH recorded in THF (4 µM in THF, 1 cm pathlength). (B) *g*-factor of chiral-diNPDH (*g*-factor = *Ι*Δ*εΙ*/*ε*). NPDH-1 and NPDH-2 refer to the first- and second-eluting enantiomers of the parent NPDH compound on chiral HPLC; chiral-diNPDH-1 and chiral-diNPDH-2 are the corresponding enantiomers of chiral-diNPDH defined in the text.

In previous work concerning extended PDI-helicenes, we have reported similar effects of amplified chiroptical response.^8,9^ When comparing this new structure with previous structures in the series it is apparent that the chiral output and chiral amplification of diNPDH are comparable in magnitude to those of the extended PDI helicene containing two [6]helicene units and three PDI units (NP3H). The novelty of diNPDH thus lies less in the absolute magnitude of the chiroptical response and more in (i) the structurally distinct architecture in which a single, electron-rich penta-perylene hub supports four PDI blades and an [E_1_]-type double carbo[6]helicene topology, (ii) the appearance of the highest |Δ*ε*| precisely in the 500–550 nm window, a region that is poorly covered by previously reported PDI helicenes (which typically peak at 400–450 nm), and (iii) the practical, gram-scale Scholl synthesis, which avoids the high-dilution photocyclizations required for the more elongated congeners. All the PDI helicenes preferentially absorb only polarized light of one handedness over a wide range of the visible spectrum (*ca.* 400–600 nm).

It should be noted that, when considering the chiraloptical properties of small molecules, the signal intensities observed for helicenes and multihelicenes are among the strongest seen. Intense chiral optical output is also often observed for certain lanthanide complexes.^29^

Cyclic voltammetry of chiral-diNPDH and *meso*-diNPDH revealed a series of reversible reductions and a single reversible oxidation event, with all events occurring at a similar voltage for both diastereoisomers (Fig. S7). The oxidation event (0.71 V *vs.* Fc/Fc^+^) is assigned to a single electron oxidation, associated with the electron rich perylene core. The reductions are divided in to two major events, with the onset of the first event occurring at −1.2 V *vs.* Fc/Fc^+^ and the second at −1.6 V *vs.* Fc/Fc^+^. Closer inspection of the reduction events of chiral-diNPDH shows a small shoulder on each reduction event, revealing that both reduction events consist of nearly simultaneous additions of electrons. By analogy to the electrochemical analysis of other similar oligo-PDI structures, we conclude that chiral-diNPDH and *meso*-diNPDH both can accept 8 electrons, 1 per imide group. This is based on the relative intensities between the oxidation event and the reduction events.^21^

## Conclusions

In conclusion, we have developed a short and practical synthetic protocol for a fully annulated chiral penta-perylene nanocarbon that features an appreciably high *g*-factor and ECD response across the majority of the visible spectrum. The ease of preparation and the resistance of diNPDH to thermally induced epimerization (no interconversion between the diastereomers observed by chiral HPLC after 2 h at 250 °C in diphenyl ether; Fig. S4) make it a suitable candidate for advanced light detecting devices and other advanced applications. These data suggest that fusing multiple [6]helicenes through an electron-rich perylene hub is a general strategy for enhancing ECD across the visible spectrum.

## Author contributions

S.·K. P. carried out the synthesis, purification, and spectroscopic characterization, and contributed to the writing of the manuscript. M. L. S. performed the quantum-chemical calculations and contributed to interpretation of the electronic structure. C. N. and M. P. conceived the study, supervised the work, and co-wrote the manuscript. All authors discussed the results and commented on the manuscript.

## Conflicts of interest

There are no conflicts to declare.

## Supplementary Material

SC-017-D6SC03791G-s001

## Data Availability

The data that support the findings of this study are available in the supplementary information (SI) of this article. Supplementary information is available. See DOI: https://doi.org/10.1039/d6sc03791g.
