# Supplementary material for: Amplified chiroptic response in a multi-helical penta-perylene structure
Source: Chem Sci. 2026 Jun 17;17(29):14352–8. doi: 10.1039/d6sc03791g (PMC13272944; doi:10.1039/d6sc03791g)
Supplement: SC-017-D6SC03791G-s001 [file SC-017-D6SC03791G-s001.pdf]

Supplementary Information (SI) for Chemical Science.
This journal is © The Royal Society of Chemistry 2026

Supporting Information

Contents

S1 General Experimental Information 2

S2 Figures and Schemes Referenced in the Manuscript 3

S3 Synthetic Procedures and Characterization Data 6

S4 1H- and ^{13}C-NMR Spectra 12

S5 High-Resolution Mass Spectrometry Data 17

S6 References 19

S1 General Experimental Information

Synthesis: All reactions were conducted in the designated oven-dried glassware. Schlenk flasks were evacuated and backfilled with argon or nitrogen three times prior to use. Anhydrous THF was obtained from a Glass Contour solvent system consisting of a Schlenk manifold with purification columns packed with activated alumina and supported copper catalyst. These solvents were dispensed from Pure-Pac™ containers purchased from Sigma-Aldrich. Anhydrous, Sure/Seal™ 1,4- dioxane was used as purchased from Sigma-Aldrich. Perylene-3,4,9,10- tetracarboxylic diimide, 1-bromoperylene-3,4,9,10-tetracarboxylicdiimide, and 1,6-dibromoperylene-3,4,9,10-tetracarboxylicdiimide were prepared using a procedure developed by Rajasingh et al.[30], Potassium carbonate, potassium acetate, and potassium phosphate were purchased from Sigma-Aldrich, ground to a fine powder using a mortar and pestle, evacuated at 200 °C for 3 h, and stored in a 200 °C oven. All remaining reagents were purchased from Sigma-Aldrich and used without additional purification, unless noted otherwise. SATCO Hi-Pro Spiral 40 W Natural Light or 55 W Bright White (2600 and 3700 lumens, respectively) compact fluorescent lamps (CFLs) were used during the oxidative photocyclizations

Purification: Automated chromatography was performed using a Teledyne Isco Combiflash® Rf200 and RediSep® normal-phase silica flash columns. Optima™ hexanes, dichloromethane, and ethyl acetate from Fisher Scientific were used as eluents. Silica plugs consisted of Silicycle SiliaFlash® P60 40-63 µm silica gel. Analytical TLC plates were cut from Silicycle SiliaPlate™ Glass Backed TLC Extra Hard Layer silica gel plates, 60 Å, 20 × 20 cm, 250 µm thickness, F-254 indicator.

Electronic Circular Dichroism: The ECD spectra were recorded using a Jasco J-810 spectropolarimeter. A 10 mm pathlength high precision cell made of Quartz SUPRASIL® from Hellma Analytics was used in the collection of the spectra.

NMR Spectroscopy: ^{1}H-NMR spectra were recorded on a Bruker 500 MHz spectrometer. ^{13}C-NMR were recorded on a Bruker 101 or 126 MHz spectrometer with complete proton decoupling. Chemical shifts for protons are reported in parts per million (ppm) downfield from tetramethylsilane (TMS) and were referenced to residual proton in the NMR solvent ($CHCl_3$, δ 7.26; $C_2H_2Cl_4$, δ 6.00. Chemical shifts for carbon are reported in ppm downfield from TMS and were referenced to the carbon resonances of the NMR solvent ($CDCl_3$, δ 77.16; $C_2D_2Cl_4$. ^{1}H-NMR data are represented as follows: chemical shift, multiplicity (s = singlet, d = doublet, dd = doublet of doublets, m = multiplet, and bm = broad multiplet), coupling constants in Hz, and integration. Most 1H- and 13C-NMR spectra were recorded at elevated temperatures (noted in K) to enhance peak resolution, particularly in the aromatic region. Peaks corresponding to the numerous aromatic carbon atoms in the reported compounds often overlap, thereby reducing the number of observed peaks.

High-Resolution Mass Spectrometry (HRMS): HRMS was conducted using a Waters XEVO G2XS instrument equipped with a UPC2 SFC inlet, electrospray (ESI) and atmospheric pressure chemical (APCI) ionization, and a QToF mass spectrometer. For large compounds, HRMS was performed using a Bruker ultrafleXtreme

MALDI-TOF/TOF with a frequency-tripled Nd:YAG laser (355 nm), linear and reflector modes, and Precursor Ion Selector and LIFT technologies for MS/MS analysis.

Quantum Mechanical Calculations: All quantum chemical calculations were performed using Jaguar, version 8.3, Schrodinger, Inc., New York, NY, 2014.9 All geometries were optimized using the B3LYP functional and the 6-31G** basis set. We calculated the absorption spectrum of the optimized geometries using the TD-DFT method that is included in the Jaguar package. We used the 6-31G** basis set in these calculations.

Voltammetry: Cyclic voltammograms in Figure 4 were recorded on a CHI600C electrochemical workstation using Ag/AgCl as the reference electrode, glassy carbon (3 mm diameter) as the working electrode, and a platinum wire as the counter electrode. Experiments were performed under argon in dichloromethane with [Bu4N][PF6] as the supporting electrolyte at a scan rate of 0.05 V/

S2 Figures and Schemes Referenced in the Manuscript

Reactions and MALDI-TOF spectra associated with table 1:

Figure S1: Reaction conditions and MALDI-TOF spectra associated with Table 1, Entry 1 and 2

Reaction:

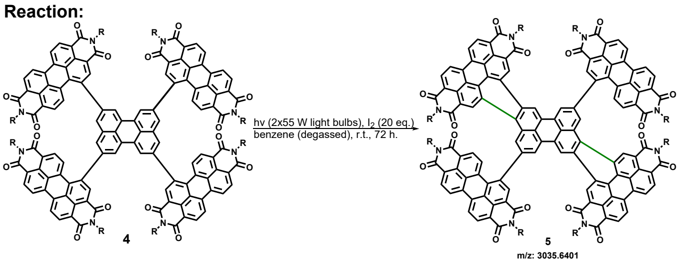

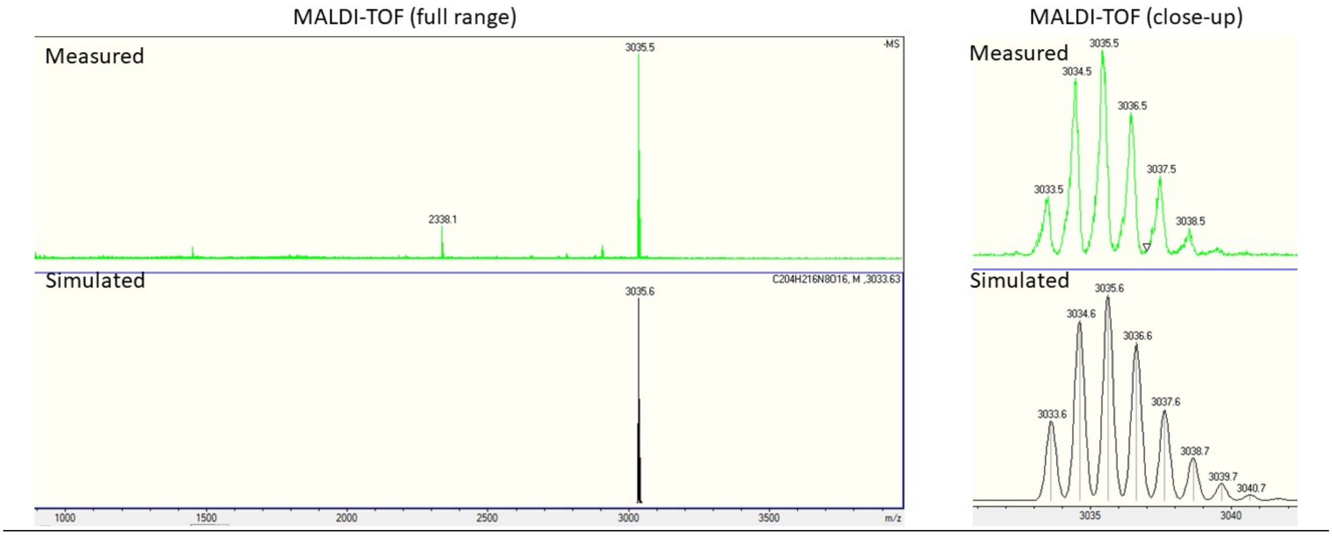

Figure S2: Reaction conditions and MALDI-TOF spectra associated with Table 1, Entry 3:

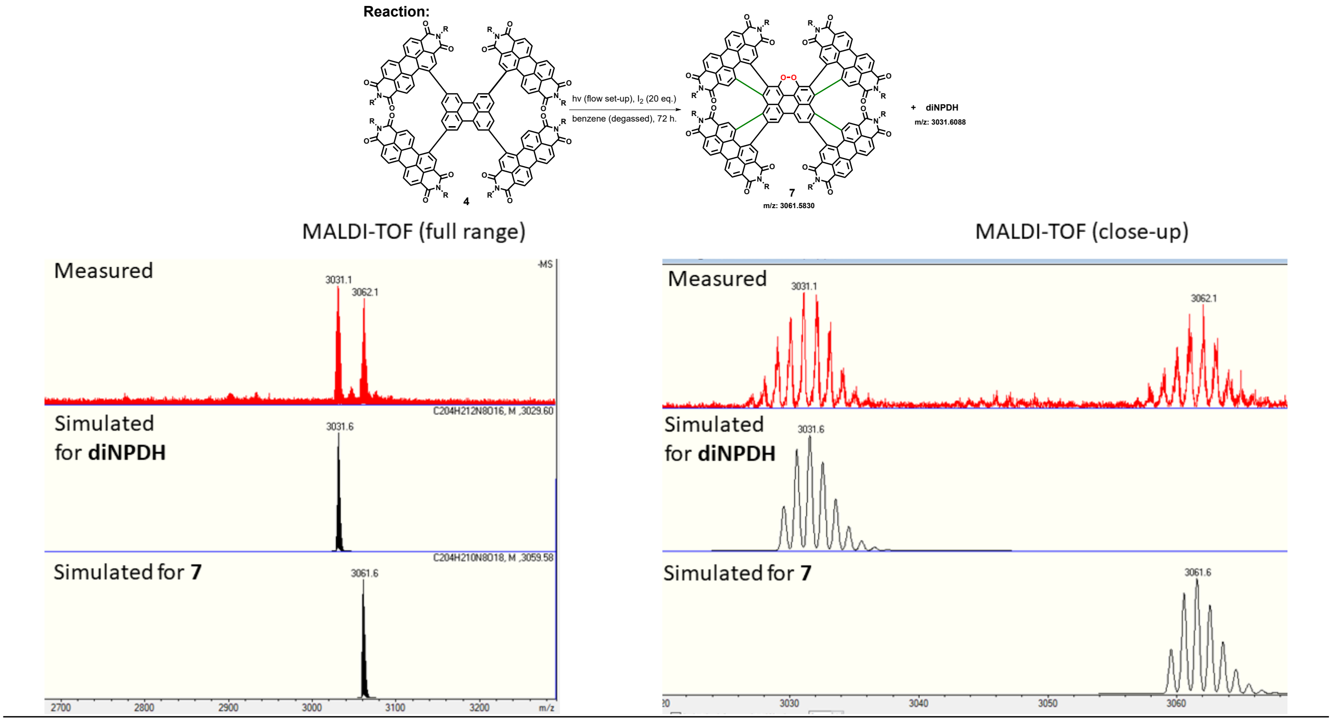

Figure S3: Reaction conditions and MALDI-TOF spectra associated with Table 1, Entry 4:

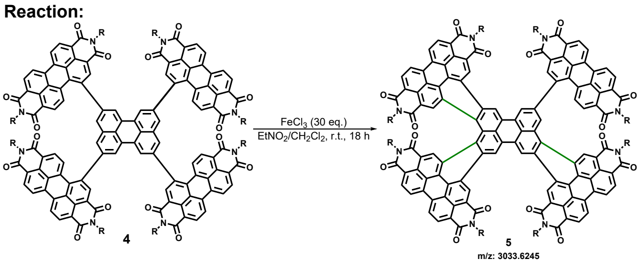

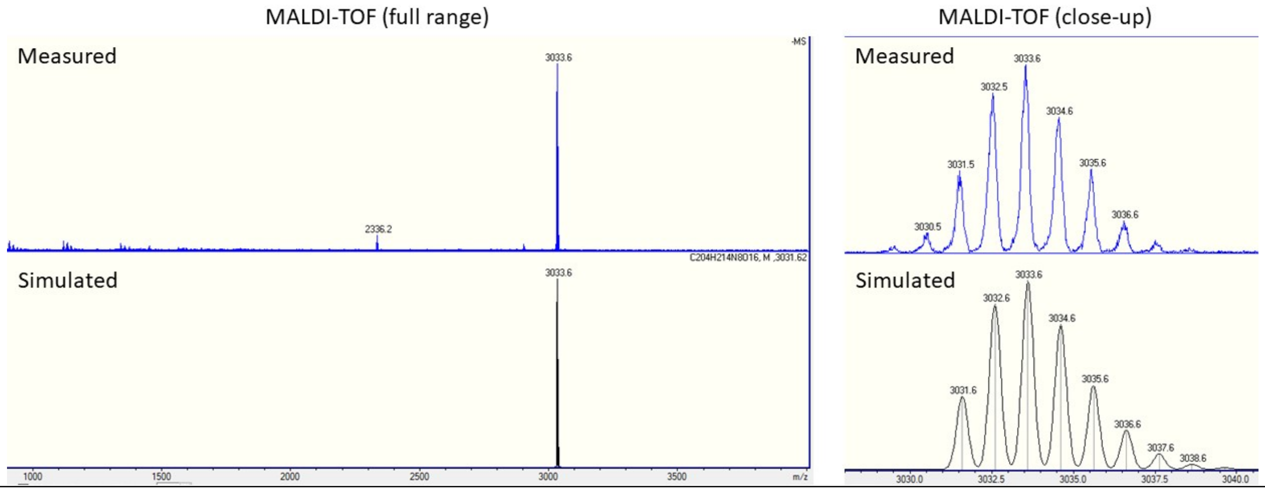
MALDI-TOF (full range)
MALDI-TOF (close-up)
Measured
Simulated
3033.6
2336.2
C204H214N8O16, M ,3031.62
3030.5
3031.5
3032.5
3034.6
3035.6
3036.6
3031.6
3032.6
3037.6
3038.6
m/z

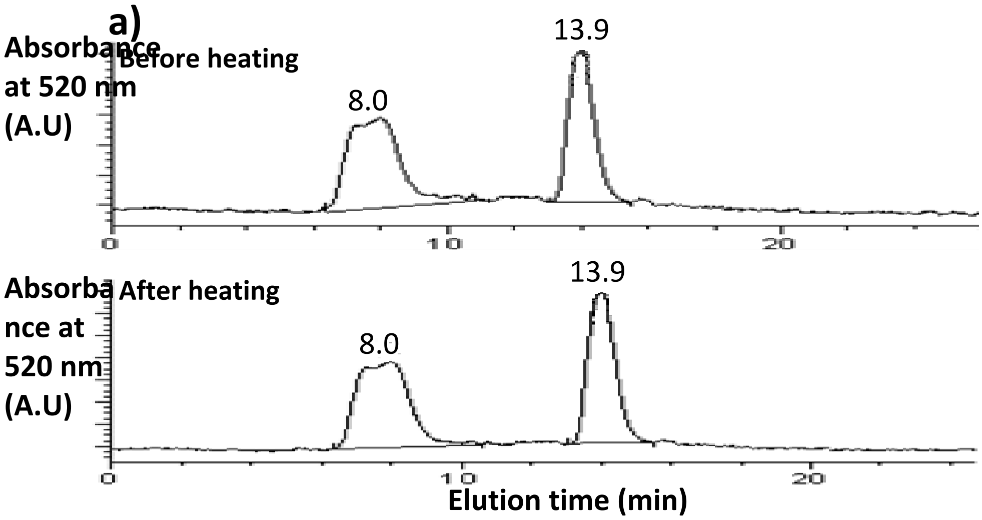
a)
Absorbance at 520 nm (A.U)
Before heating
8.0
13.9
Absorbance at 520 nm (A.U)
After heating
8.0
13.9
Elution time (min)

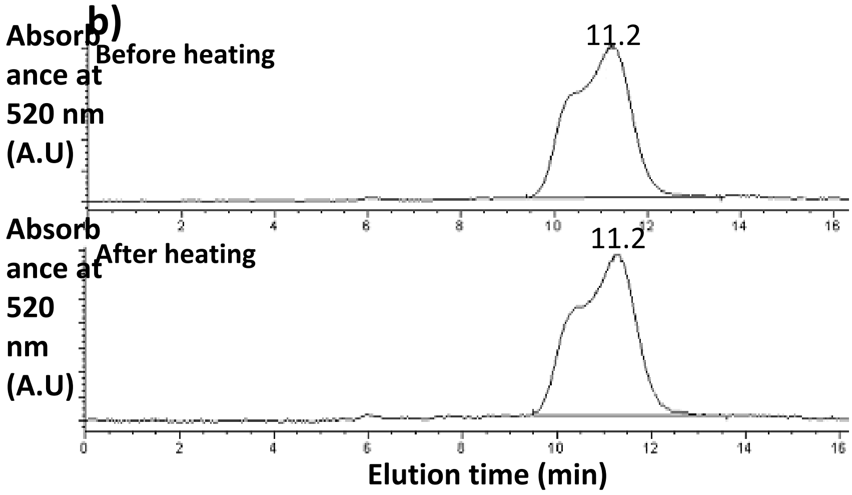

Figure S4: Chiral HPLC chromatograms of **chiral-diNPDH** (**A**) and **meso-diNPDH** (**B**) before and after heating for 2 hours at 250 ° C in diphenylether. The peak broadening observed is attributed to large quantity of diphenylether present in the samples. **The HPLC conditions are described in the caption of Figure S7.**

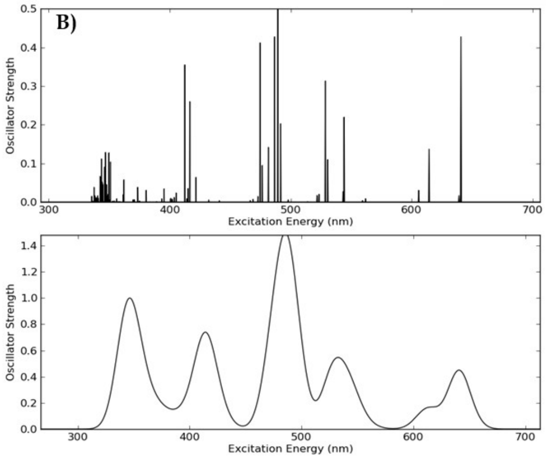

Figure S5: *top*) TD-DFT (B3LYP/6-21G**) calculated oscillator strength (40 strongest oscillators) for **chiral-diNPDH**. *bottom*) all oscillators set to 20 nm FWHM.

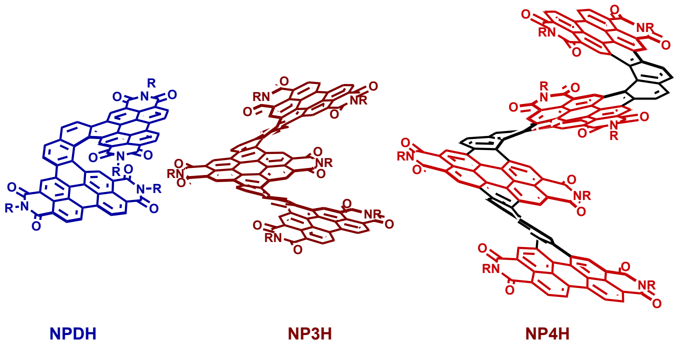

Figure S6: Structure of series of PDI-helicenes.

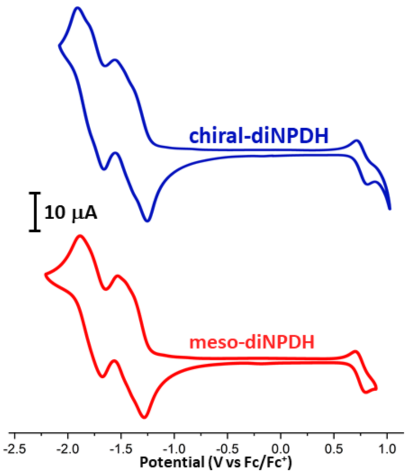

Figure S7: Cyclic voltammograms of **meso-diNPDH** and **chiral-diNPDH** (~1 mM, 50 mV s^{-1} scan rate) in argon spared dichloromethane with 0.1 M $[Bu_4N][PF_6]$ as the supporting electrolyte.

S3 Synthetic Procedures and Characterization Data

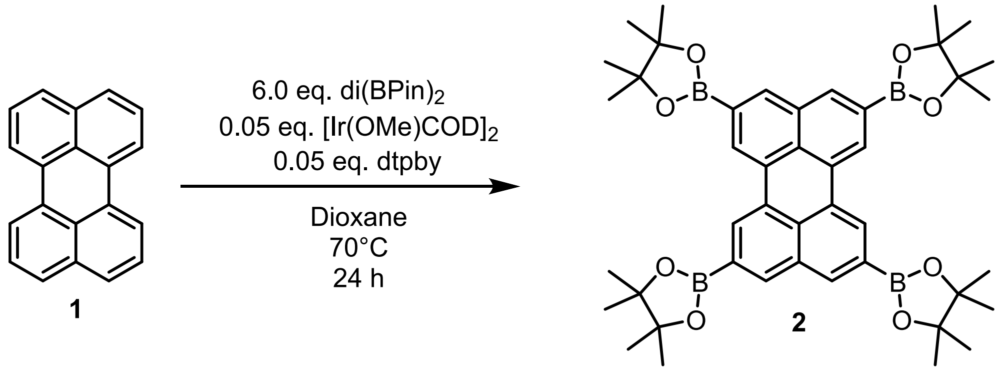

Procedure: To a flame dried Schlenk flask, fitted with a magnetic stir bar, $[Ir(OMe)COD]_2$ (0.131 g, 0.198 mmol, 0.05 eq.), 4,4'-di-*tert*-butyl-2,2'-bipyridine (0.053 g, 0.198 mmol, 0.05 eq.), Bis(pinacolato)diboron (6.04 g, 23.8 mmol, 6.0 eq.) and perylene (**1**) (1.00 g, 3.96 mmol, 1.00 eq.) was added. The Schlenk flask

was sealed with a rubber septum, and cycled between a nitrogen atmosphere and vacuum three times. To the nitrogen filled flask was added degassed anhydrous dioxane (79 mL). The reaction mixture was heated at 80 ˚C for 24 hours. The contents of the Schlenk flask was transferred to a round bottomed flask, and all volatiles were removed under reduced pressure. Acetone (50 mL) was added to the round bottomed flask, and the solids were suspended by sonication for 5 minutes. The suspension is filtered through a glass sintered funnel, and the solids washed with room tempered acetone (200 mL), leaving **2** as a yellow solid.

The protocol is a modification of a previously reported literature protocol.[31]

Yield: 2.88 g, 3.81 mmol, 96%

^{1}H NMR: (500 MHz, $CDCl_3$, 300K) δ 8.62 (s, 4H), 8.25 (s, 4H), 1.43 (s, 48H)

^{13}C NMR: (126 MHz, $CDCl_3$) δ 137.1, 133.4, 132.1, 130.5, 126.2, 126.7, 84.2, 25.0

HR-MS: ($M+H^+$) calculated for $C_{44}H_{57}B_4O_8$ = 757.4453 m/z; found 757.4456

All data in accordance with what has previously been reported.[31]

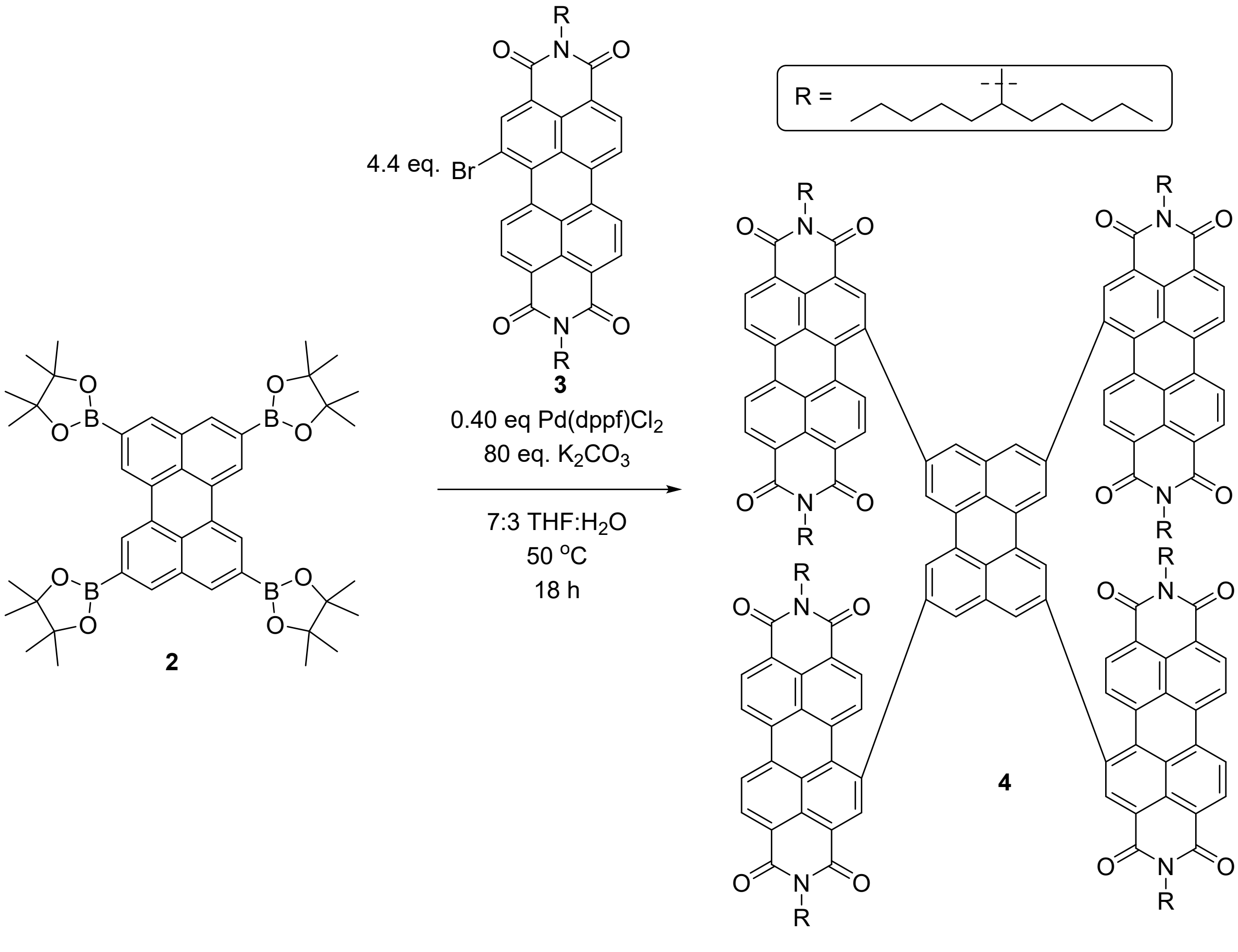

Procedure: 2,5,8,11-tetrakis-Bpin-perylene (**2**) (0.331 g, 0.438 mmol, 1.00 eq.), **3** (1.50 g, 1.93 mmol, 4.4 eq), Pd(dppf)Cl_2 (0.128 g, 0.175 mmol, 0.40 eq.) and K_2CO_3 (4.85 g, 35.1 mmol, 80.0 eq.) was added to a Schlenk flask, fitted with a magnetic stir bar. The flask was put under vacuum and backfilled with nitrogen three times. To the nitrogen filled Schlenk flask was added degassed THF (12.3 mL) and degassed demineralized water (5.3 mL). While maintaining a nitrogen atmosphere the reaction mixture was heated at 50˚C for 18 hours. To the completed reaction was added dichloromethane (50 mL) and the organic layers were washed three times with water (50 mL) and finally brine (50 mL). The organic layer was dried with Na_2SO_4 and the volatiles removed under reduced pressure. The obtained residue was purified with automated flash column chromatography using a gradient of dichloromethane:EtOAc (9:1) and hexanes to obtain the target product.

Yield: 1.20 g, 0.395 mmol, 88%

^{1}H-NMR (500 MHz, $C_2Cl_4D_2$, 370 K) δ 8.26-7-92 (m, 24 H), 7.80-7.52 (m, 8H) 7.42-7.04 (m, 4H), 4.65-4.35 (m, 8H), 1.75 1.40 (m, 16H), 1.36-1.12 (m, 16H), 0.87-0.39 (m, 96 H), 0.38-0.00 (m, 48 H).

^{13}C-NMR (500 MHz, $C_2Cl_4D_2$, 370 K) δ 164.0, 163.7, 142.6, 142.5, 142.4, 140.5, 135.9, 135.9, 135.2, 135.1, 135.1, 135.0, 135.0, 134.5, 134.4, 134.4, 134.3, 133.6, 133.6, 133.5, 133.1, 133.0, 132.9, 131.2, 131.1, 130.8, 130.2, 129.4, 128.9, 128.5, 128.4, 128.3, 128.0, 128.0, 127.6, 124.1, 123.6, 123.5, 123.3, 122.8, 122.7, 122.2, 55.1, 54.9, 53.5, 32.6, 32.5, 31.7, 31.7, 26.7, 26.6, 22.4, 22.4, 13.9, 13.8.

HR-MS (MALDI-TOF): calculated for $[C_{204}H_{220}N_8O_{16}]^+$ is 3039.6160 m/z, found 3039.6708

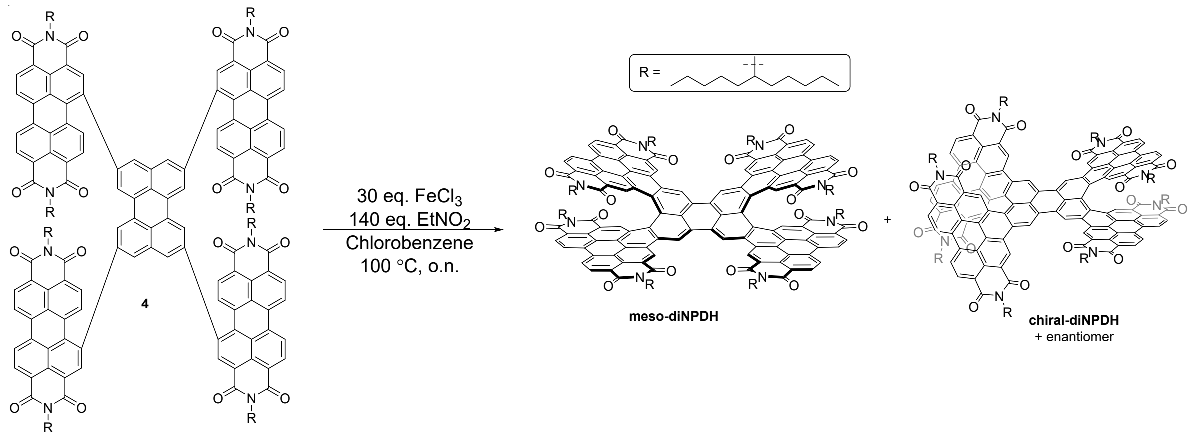

Procedure: **4** (1.2 g, 0.395 mmol, 1.00 eq.) was dissolved in chlorobenzene (49.0 mL) and the solution was degassed with nitrogen for 15 minutes. Anhydrous iron (III)chloride (1.92 g, 11.8 mmol, 30.0 eq.) was dissolved in nitroethane (3.95 ml, 55.2 mmol) and added dropwise to the solution of **4**. The reaction mixture was heated to 100 ˚C and left stirring overnight. To the completed reaction was added ethyl acetate and the organic layers was washed three times with water (75 mL) and finally brine (100 mL). The organic fractions were dried with Na_2SO_4, and the volatiles removed under reduced pressure.

Purification: As the products are inseparable on silica gel, preparative HPLC was used for purification. HPLC purification was performed on a Waters Prep150 instrument equipped with a UV-vis detector, an automated fraction collector, and a Nacalai Tesque COSMOSIL Buckyprep column (20 mm I.D. x 250 mm, 5 µm). 16.8 mg of the crude reaction mixture was dissolved in 4.0 mL DCM and 6.0 mL hexanes. The solution was passed through a PTFE syringe filter, and 7.5 mL injected in to preparative HPLC. Using an isocratic eluent of DCM (40 vol%) in hexanes and a flow rate of 19 mL/min **chiral-diNPDH** (5.5 mg, 44 % yield) and **meso-diNPDH** (3.6 mg, 28 %) were separated and isolated (See Figure S8).

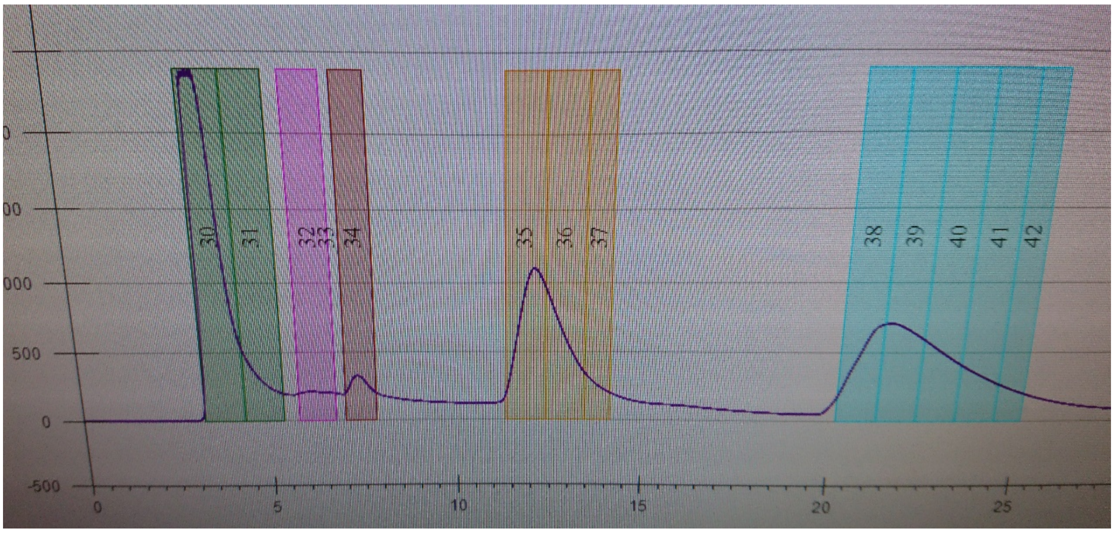

Figure S8: Chromatogram of the preparative HPLC run for the separation of the diastereomers of diNPDH. Meso-diNPDH elutes as the blue fraction. Interestingly both the green and yellow marked areas of the chromatogram corresponds to chiral diNPDH. The fact that it elutes as two separate fractions is attributed to the formation of aggregates at high concentration. Aggregates has also been observed by ^{1}H-NMR in saturated solutions of $CDCl_3$, $C_2D_2Cl_4$ and benzene-d6.

Chiral HPLC: The sample is dissolved in 50 % toluene in hexanes and injected onto a CHIRALPAK® IA3 column. The eluent system used was a linear gradient of 50 to 75 % toluene in hexanes over 20 minutes with a flow rate of 1.0 mL/min. Chromatograms are shown below (Figure S9).

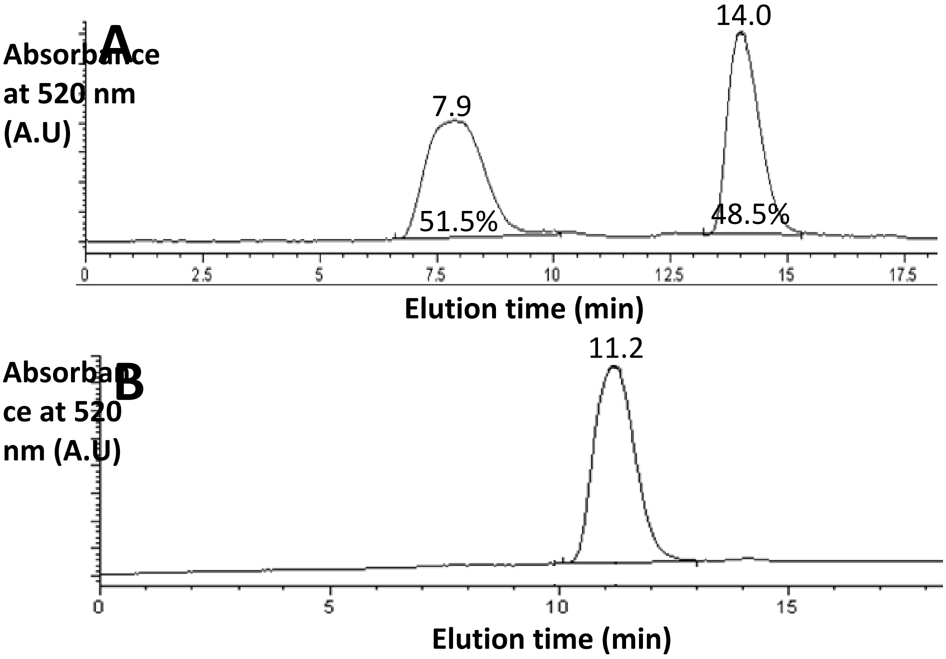

Figure S9: A) Chromatogram of **Chiral-diNPDH**. **B**) Chromatogram of **meso-diNPDH**. Identical HPLC conditions are used for both samples.

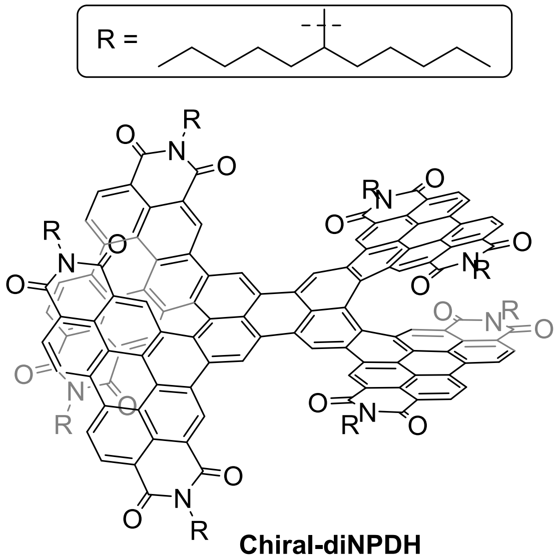

^{1}H-NMR: (500 MHz, $C_2Cl_4D_2$, 370 K): δ 10.96 (s, 4H), 10.93 (s, 4H), 9.41 (d, *J* = 8.05 Hz, 4H), 9.33-9.27 (m, 8H), 8.94 (d, *J* = 7.78 Hz, 4H), 5.58-5.50 (m, 4H), 4.60-4.48 (m, 4H), 2.70-2.48 (m, 8H), 2.25-2.10 (m, 8H), 1.70-0.74 (m, 160 H).

^{13}C NMR (126 MHz, Toluene-*d8*) δ 164.4, 164.1, 163.4, 162.8, 137.3, 136.9, 136.8, 133.9, 133.7, 132.5, 130.0, 129.8, 129.3, 128.7, 128.7, 127.7, 126.9, 126.6, 126.2, 125.8, 125.0, 125.0, 124.9, 124.2, 123.6, 123.1, 123.1, 122.4, 121.4, 120.2, 55.3, 53.9, 33.2, 33.0, 32.9, 32.1, 32.1, 31.8, 31.6, 31.3, 31.3, 29.7, 29.6, 29.3, 27.2, 26.1, 26.0, 22.7, 22.7, 22.5, 22.4, 22.1.

HR-MS (MALDI-TOF): calculated for $[C_{204}H_{212}N_8O_{16}]^+$ is 3031.6082 m/z, found 3031.5944.

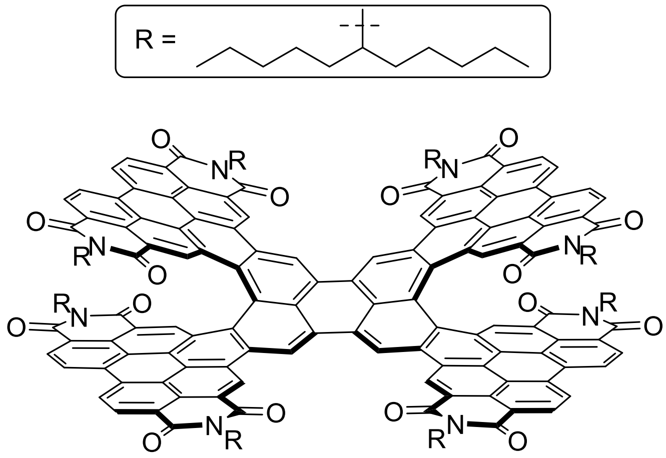

meso-diNPDH

^{1}H NMR (500 MHz, $C_2Cl_4D_2$, 370K) δ 11.10 (s, 8H), 9.38 (d, *J* = 7.86, 4H), 9.29 (d, *J* = 7.86, 4H), 9.26 (d, *J* = 7.81, 4 H), 8.91 (d, *J* = 7.81, 4H), 8.81 (s, 4H), 5.66-5.56 (m, 4H), 4.68-4.45 (m, 4H), 2.76-2.59 (m, 8H), 2.38-2.19 (m, 8H), 1.89-0.74 (m, 160 H).

^{13}C NMR (126 MHz, $C_2Cl_4D_2$, 370K) δ 163.7, 162.8, 134.0, 133.9, 132.8, 130.7, 130.4, 129.7, 128.6, 128.4, 128.3, 128.2, 127.1, 126.5, 126.3, 126.2, 125.9, 125.0, 124.6, 123.5, 123.4, 122.3, 121.1, 120.3, 74.3, 74.1, 73.8, 55.7, 54.3, 33.0, 32.1, 32.0, 31.9, 31.6, 29.7, 29.3, 27.2, 27.1, 26.4, 26.2, 22.7, 22.7, 22.5, 22.3, 14.1, 14.0, 14.0.

Due to overlap of the resonances only 47 carbons out of the expected 52 are observed.

HR-MS (MALDI-TOF): calculated for $[C_{204}H_{212}N_8O_{16}]^+$ is 3031.6082 m/z, found 3031.5787.

S4 1H- and ^{13}C-NMR Spectra

^{1}H-NMR of **2** ($CDCl_3$, 300K)

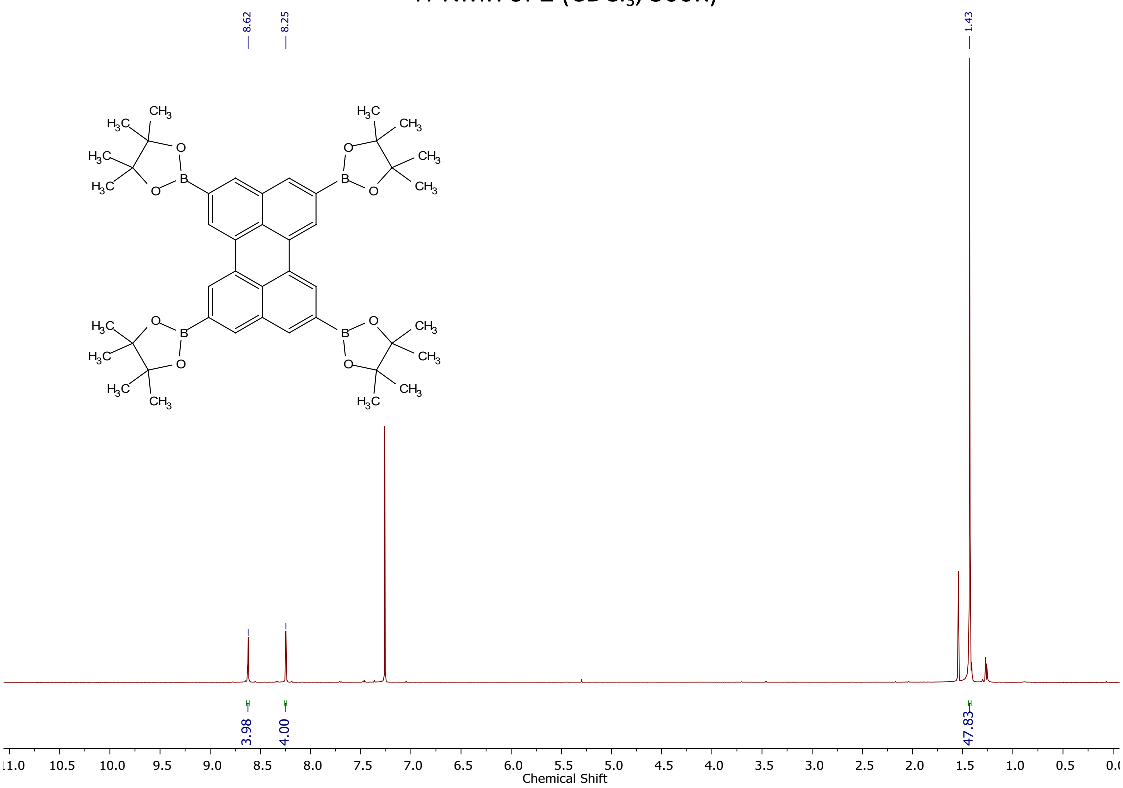

^{13}C-NMR of **2** ($CDCl_3$, 300 K)

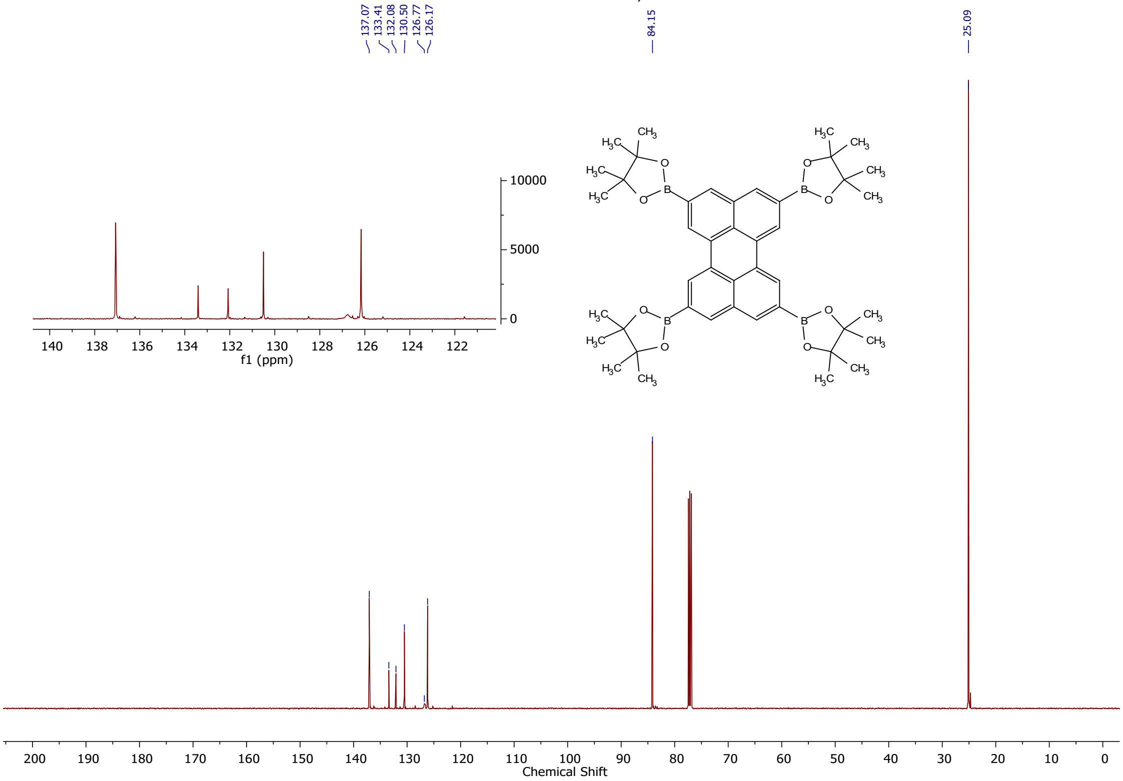

^{1}H NMR of **4** ($C_2D_2Cl_4$, 370 K)

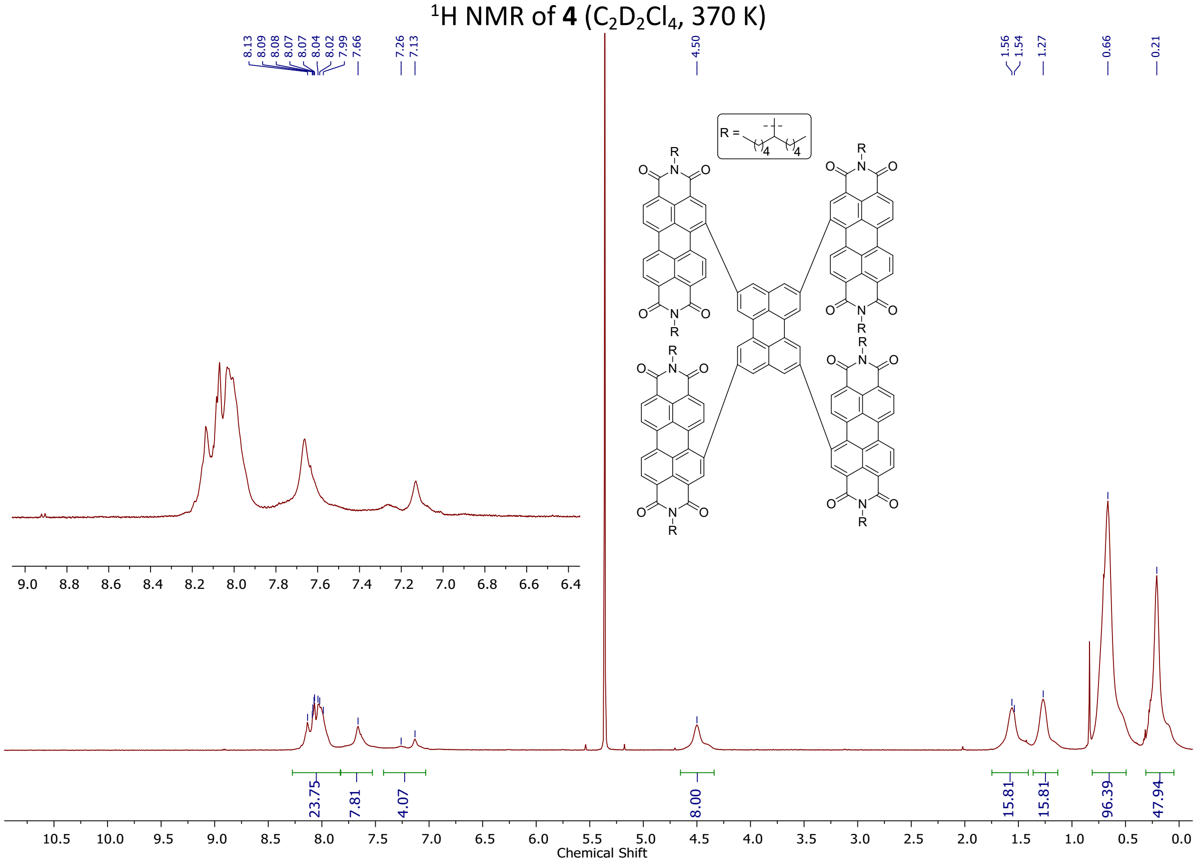

^{13}C NMR of **4** ($C_2D_2Cl_4$, 370 K)

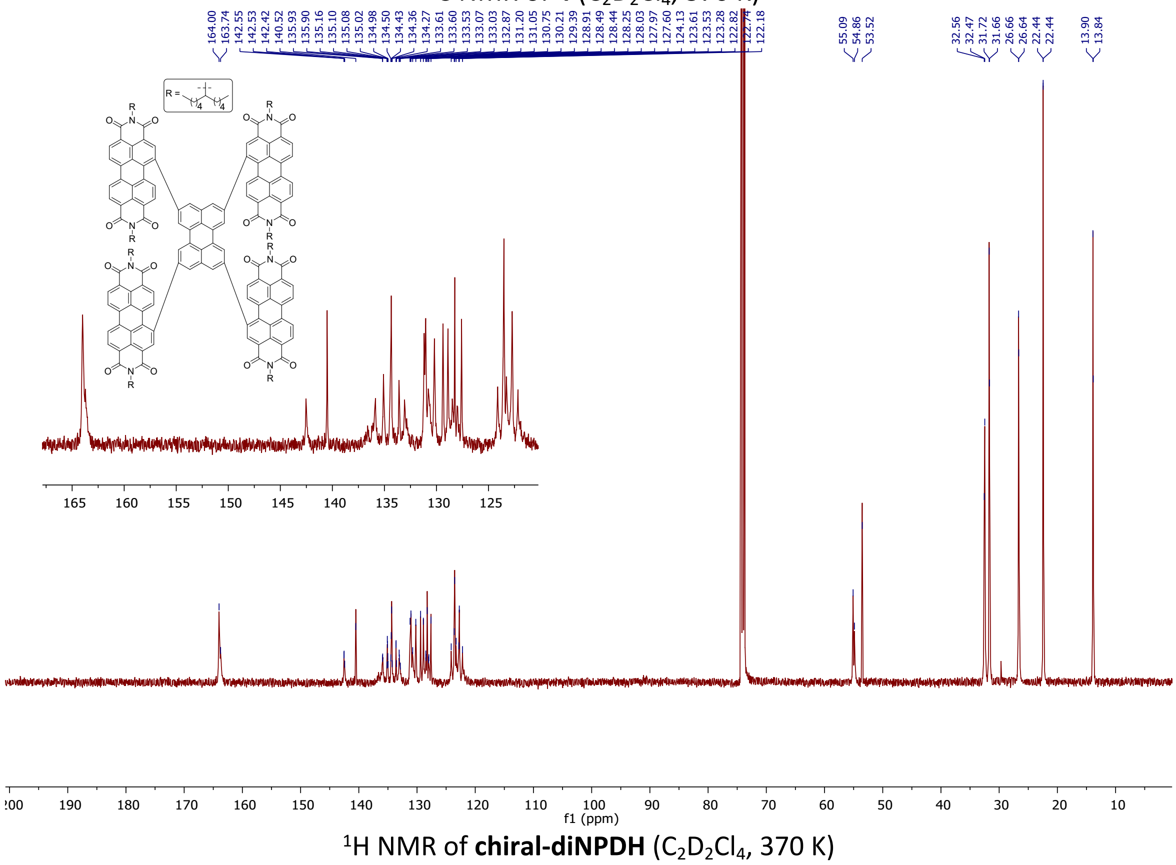

^{1}H NMR of **chiral-diNPDH** ($C_2D_2Cl_4$, 370 K)

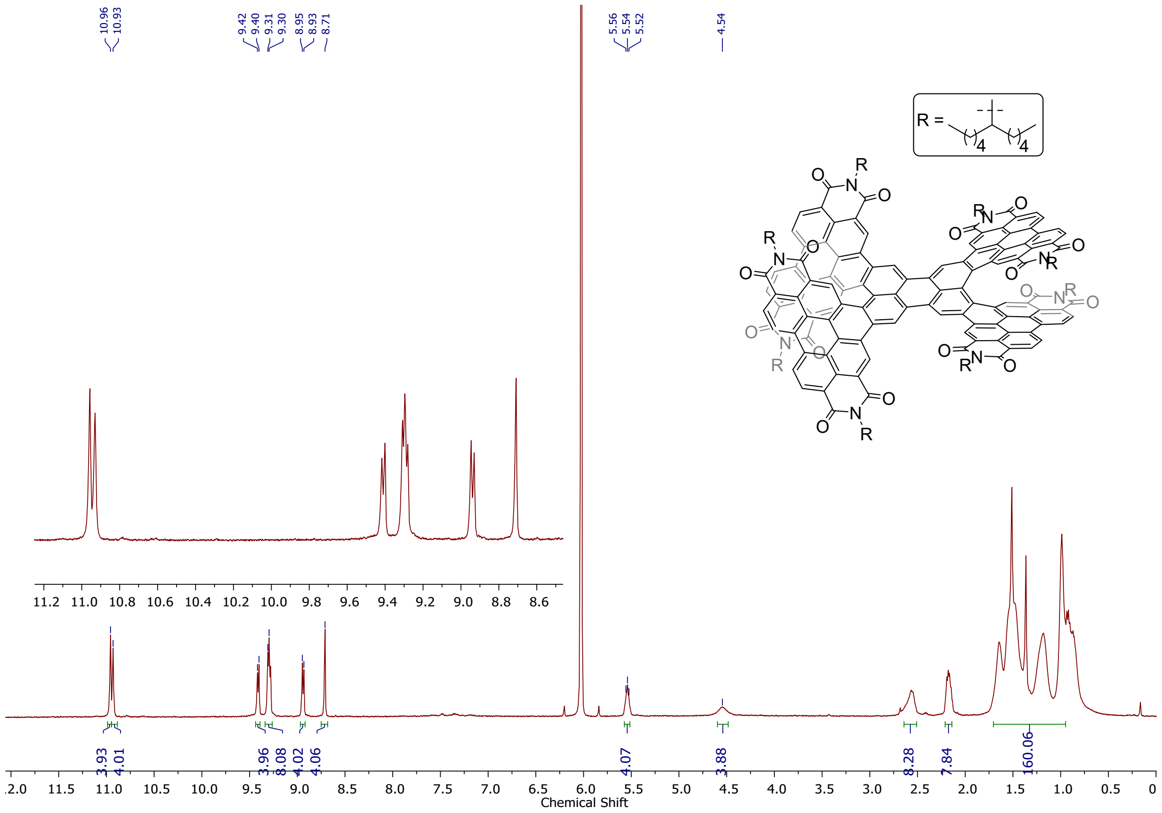

^1H-^1H COSY of **chiral-diNPDH** ($C_2D_2Cl_4$, 370 K). The relevant COSY signals are highlighted in the insert.

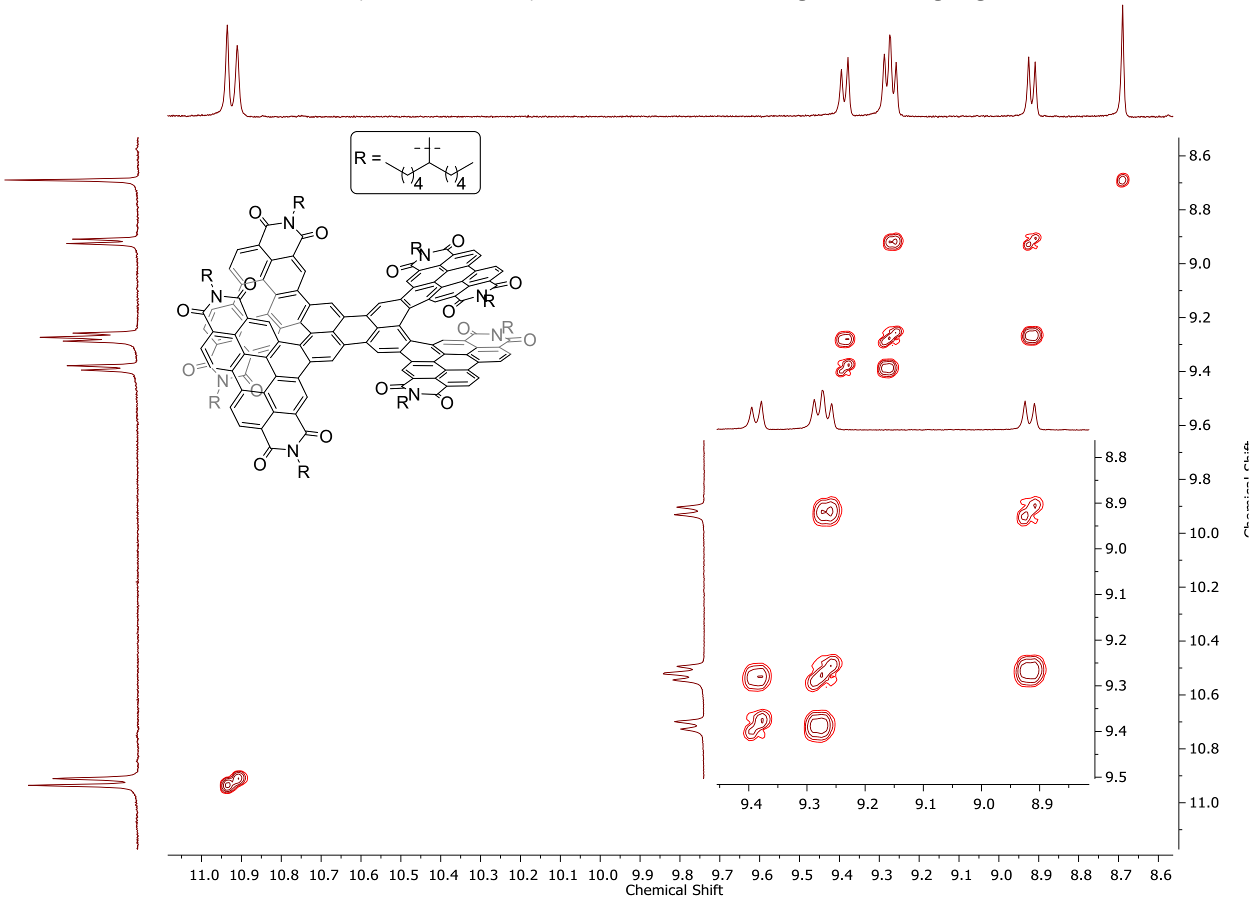

^{13}C-NMR of c**hiral-diNPDH** (toluene-*d6*, 370 K)

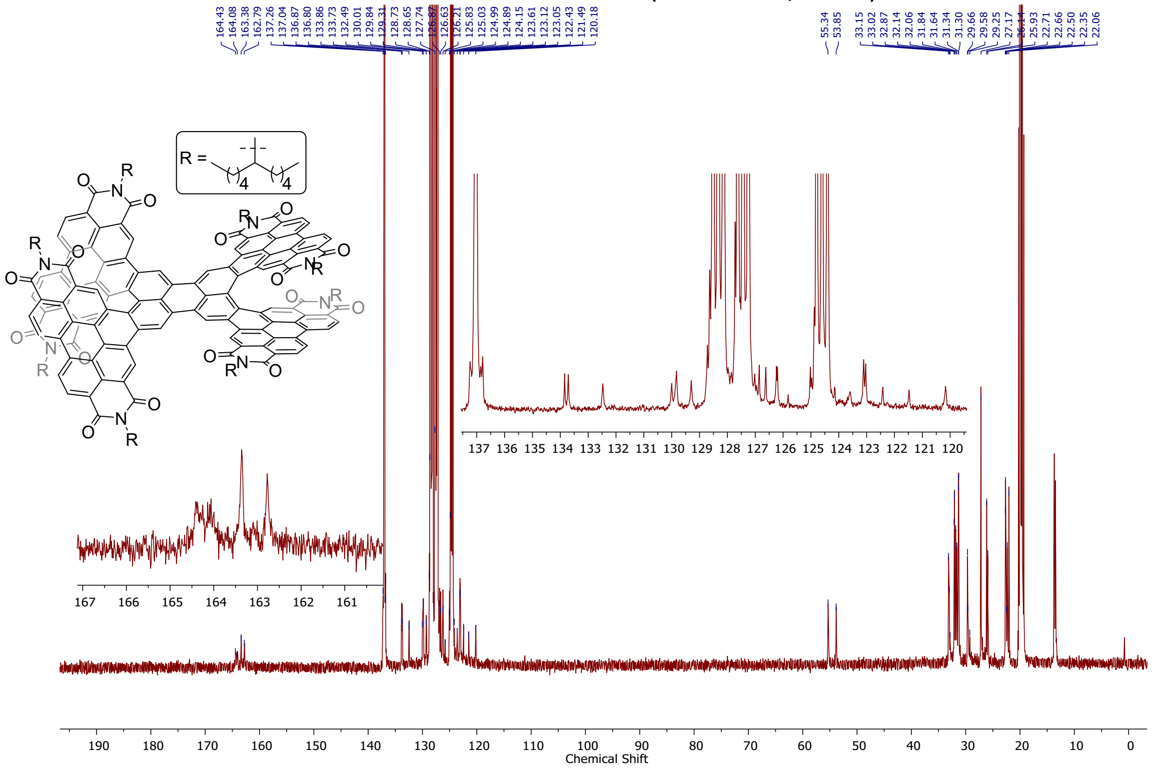

^{1}H-NMR of **meso-diNPDH** ($C_2D_2Cl_4$, 370 K)

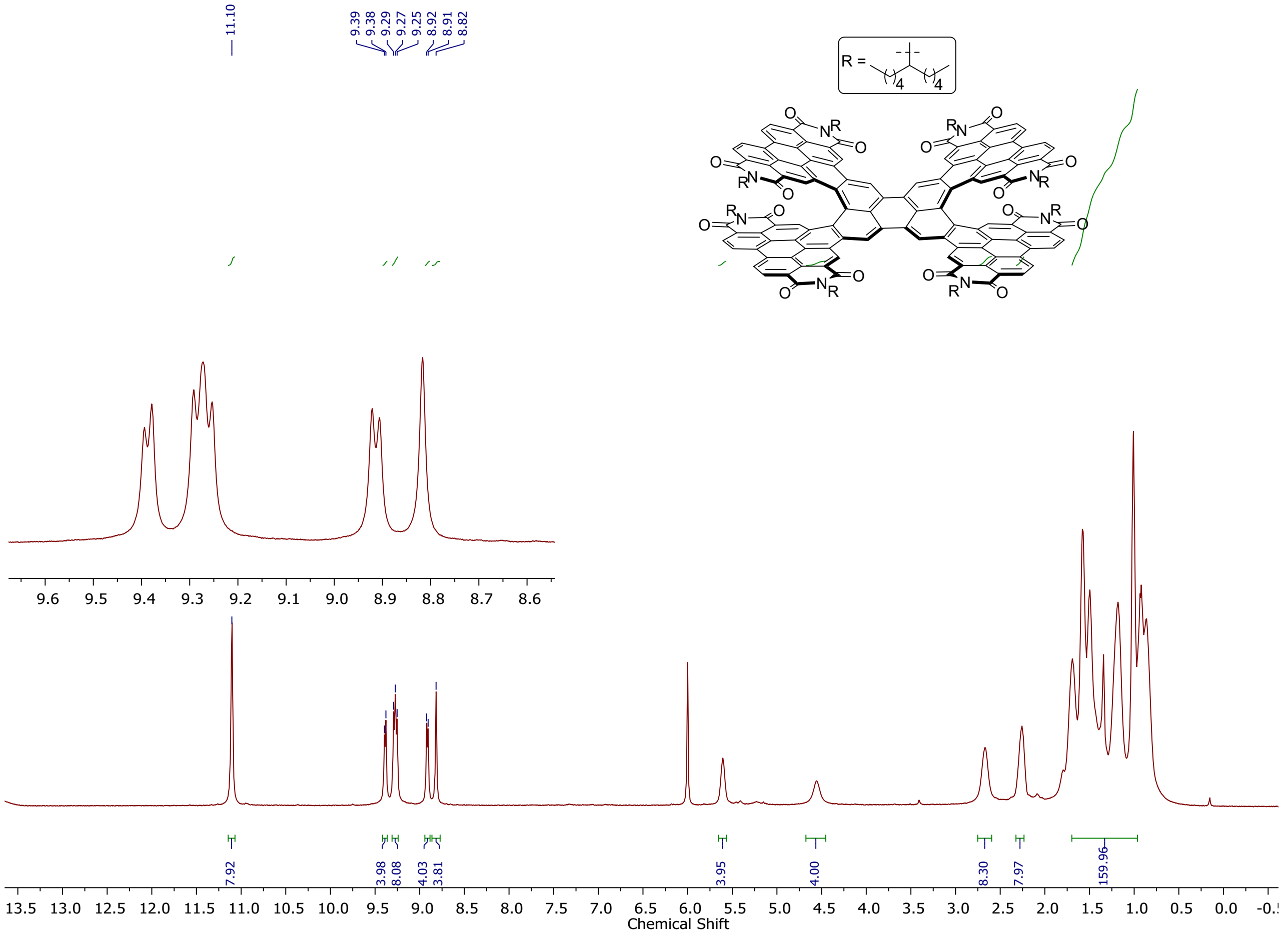

^{1}H-^{1}H COSY of **meso-diNPDH** ($C_2D_2Cl_4$, 370 K). The relevant COSY signals are highlighted in the insert.

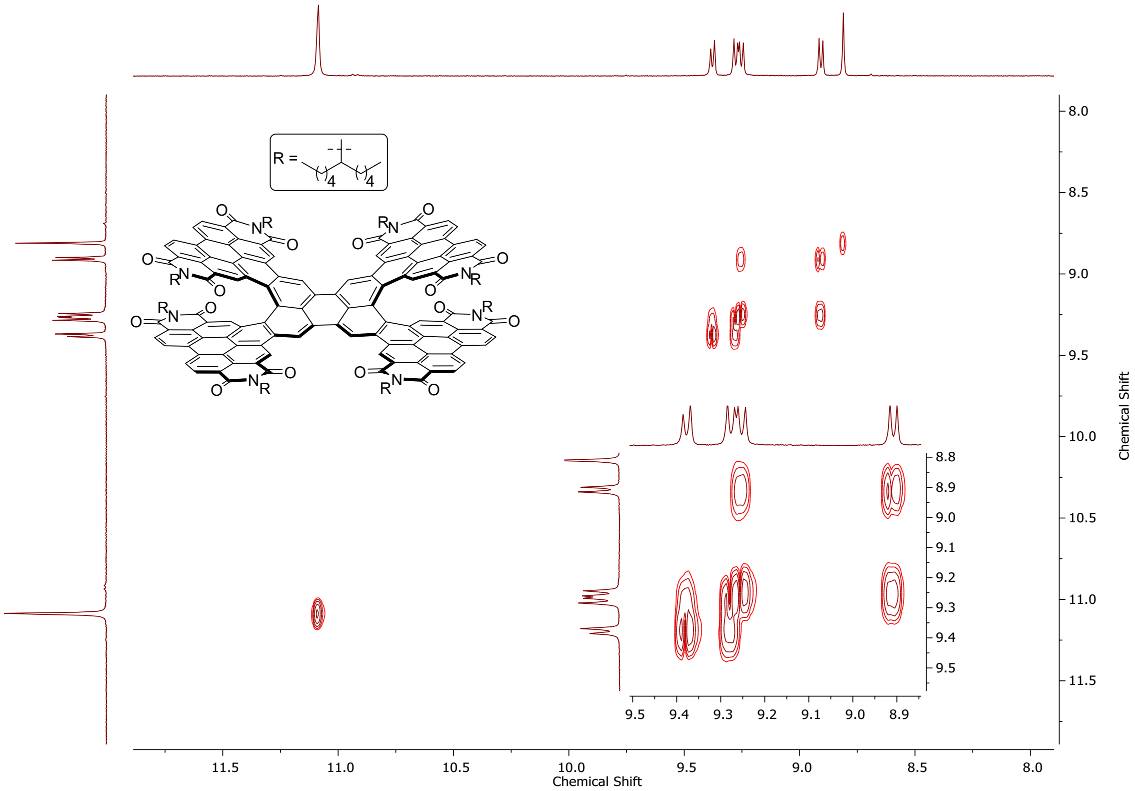

^{13}C-NMR of **meso-diNPDH** ($C_2D_2Cl_4$, 370 K)

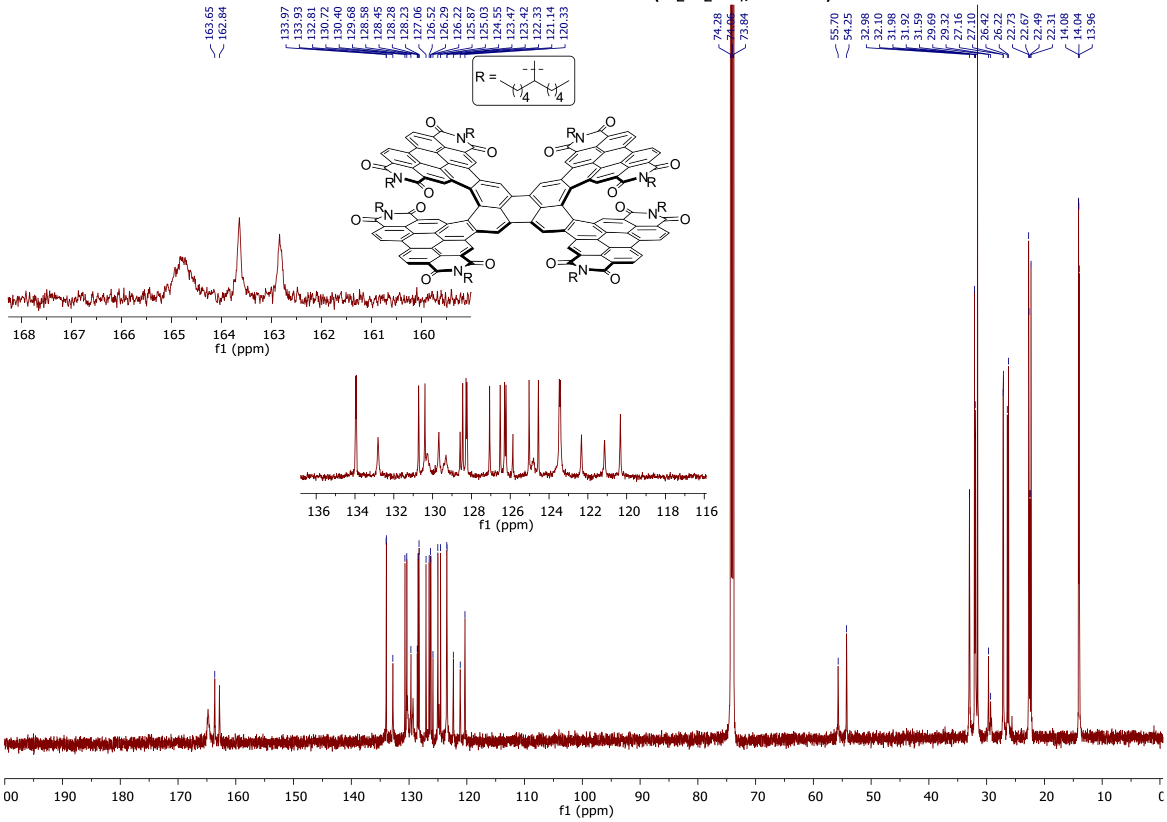

S5 High-Resolution Mass Spectrometry Data

HR-MS of **2**

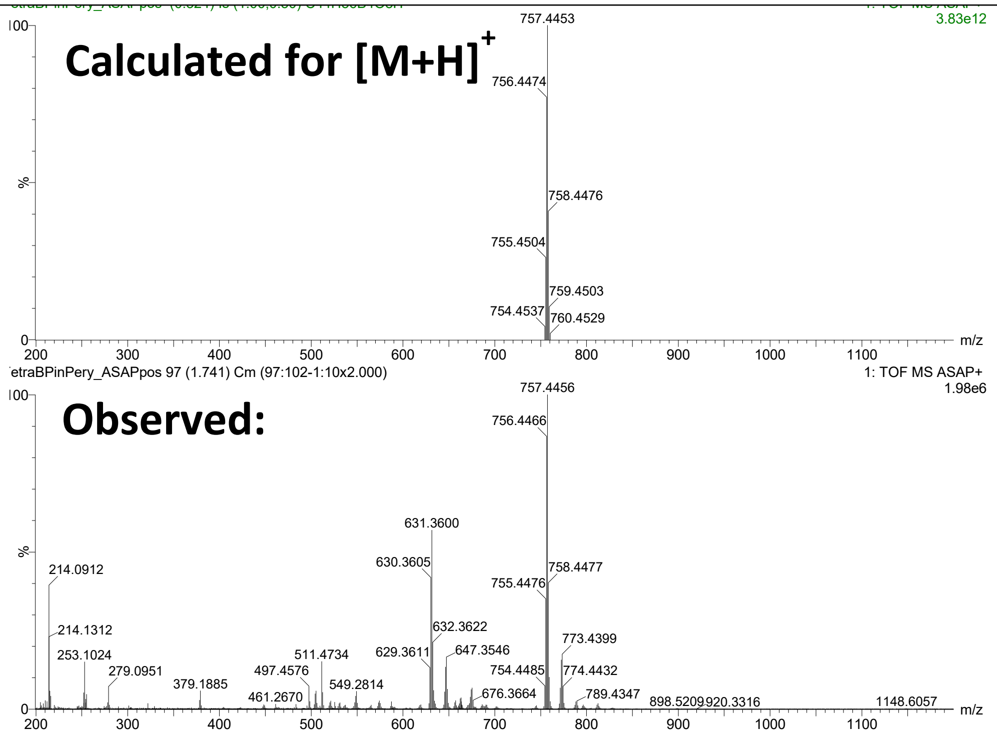

HR-MS of **2**:

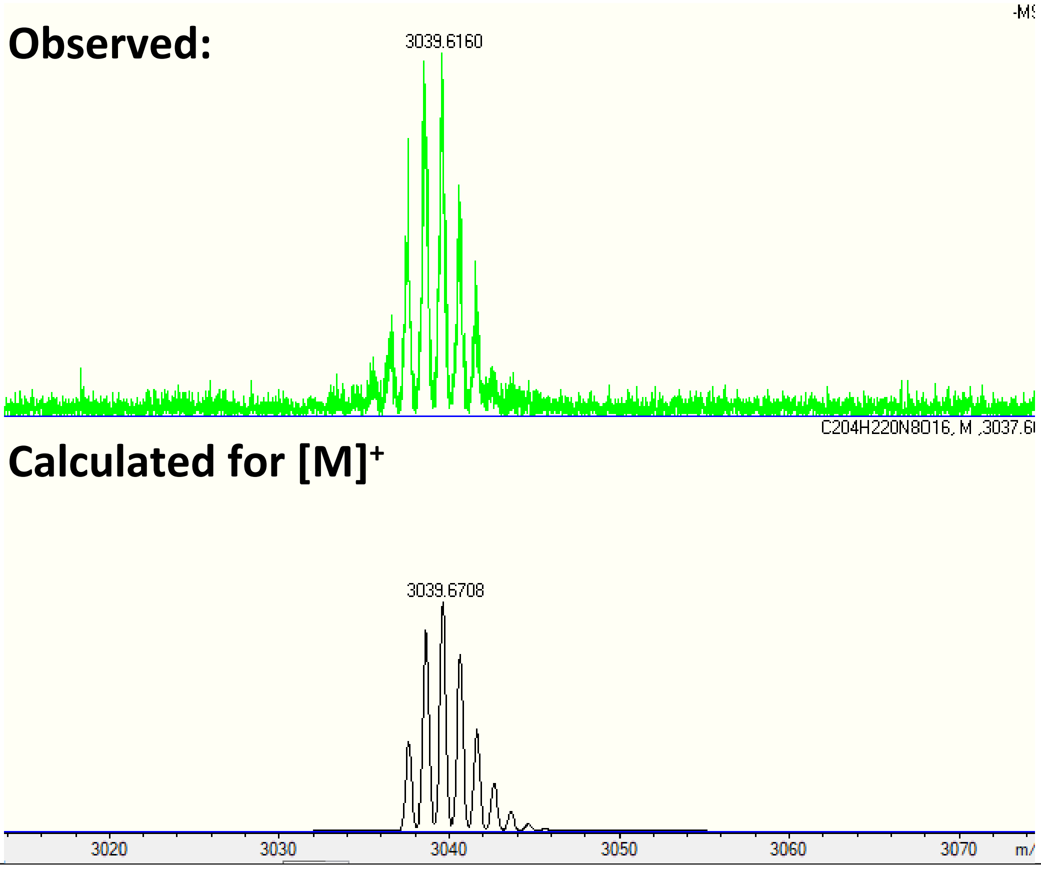

HR-MS of **chiral-diNPDH**:

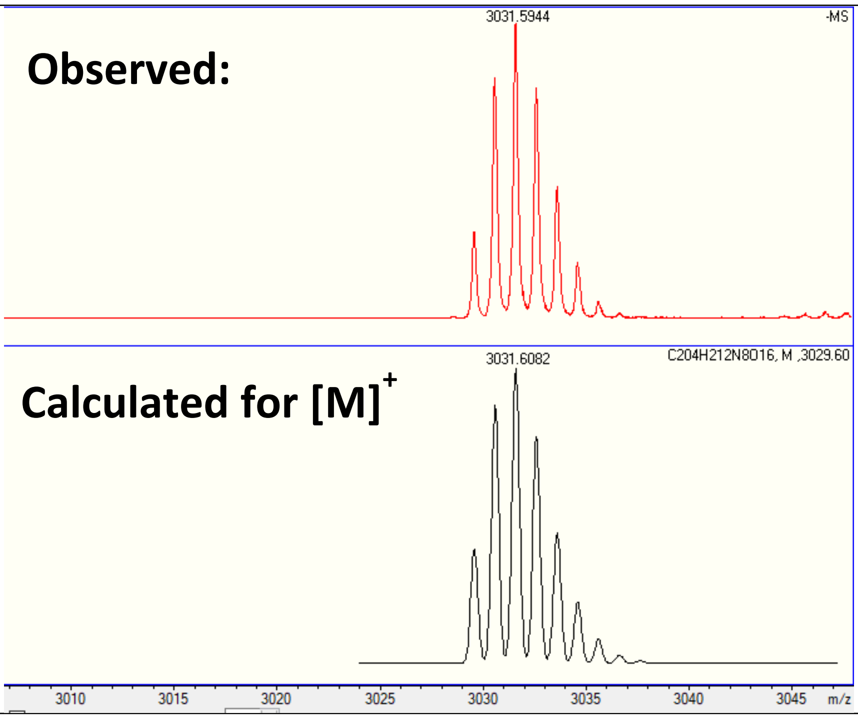

HR-MS of **Meso-diNPDH**:

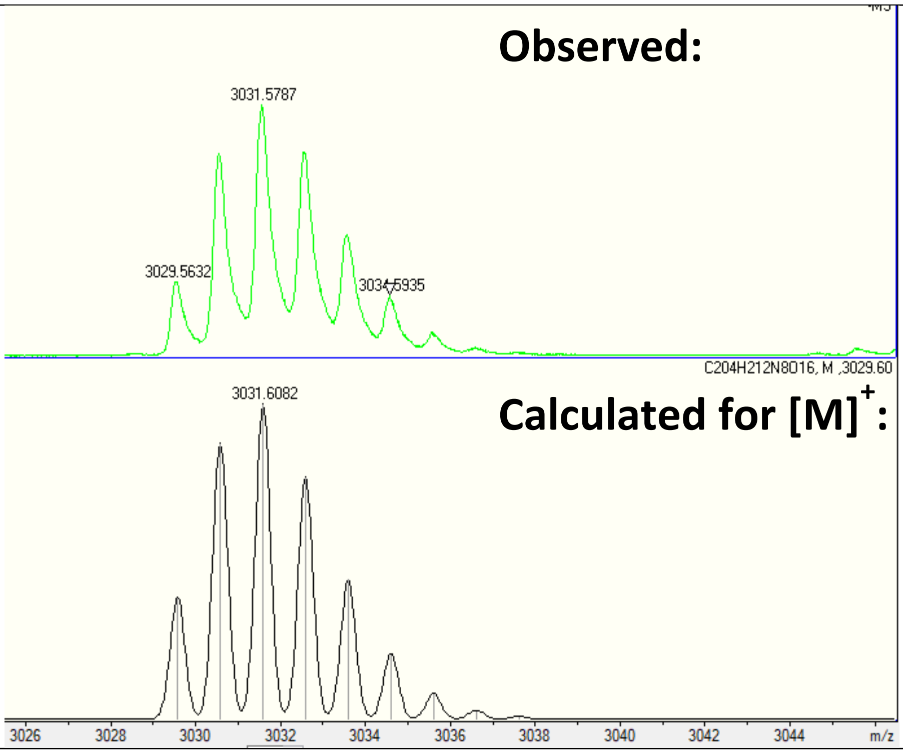

S6 References

[30] Rajasingh, P.; Cohen, R.; Shirman, E.; Shimon, L. J. W.; Rybtchinski, B. Selective Bromination of Perylene Diimides under Mild Conditions. *J. Org. Chem.* **2007**, *72* (16), 5973–5979. https://doi.org/10.1021/jo070367n.

[31] Coventry, D. N.; Batsanov, A. S.; Goeta, A. E.; Howard, J. A. K.; Marder, T. B.; Perutz, R. N. Selective Ir-Catalysed Borylation of Polycyclic Aromatic Hydrocarbons: Structures of Naphthalene-2,6-Bis(Boronate), Pyrene-2,7-Bis(Boronate) and Perylene-2,5,8,11-Tetra(Boronate) Esters. *Chem. Commun.* **2005**, No. 16, 2172–2174. https://doi.org/10.1039/b501778e.
